# Twenty-first-century projections of shoreline change along inlet-interrupted coastlines

**DOI:** 10.1038/s41598-021-93221-9

**Published:** 2021-07-07

**Authors:** Janaka Bamunawala, Roshanka Ranasinghe, Ali Dastgheib, Robert J. Nicholls, A. Brad Murray, Patrick L. Barnard, T. A. J. G. Sirisena, Trang Minh Duong, Suzanne J. M. H. Hulscher, Ad van der Spek

**Affiliations:** 1 Department of Water Engineering and Management, University of Twente, P.O. Box 217, 7500 AE Enschede, The Netherlands; 2 IHE Delft Institute for Water Education, P.O. Box 3015, 2601 DA Delft, The Netherlands; 3 Harbour, Coastal and Offshore Engineering, Deltares, P.O. Box 177, 2600 MH Delft, The Netherlands; 4 Tyndall Centre for Climate Change Research, University of East Anglia, Norwich, NR4 7TJ UK; 5 Division of Earth and Ocean Sciences, Nicholas School of the Environment, Center for Nonlinear and Complex Systems, Duke University, Box 90229, Durham, NC 27708-0229 USA; 6 Pacific Coastal and Marine Science Center, United States Geological Survey, 2885 Mission Street, Santa Cruz, CA 95060 USA; 7 Applied Morphodynamics, Deltares, P.O. Box 177, 2600 MH Delft, The Netherlands; 8 Department of Physical Geography, Faculty of Geosciences, Utrecht University, P.O. Box 80115, 3508 TC Utrecht, The Netherlands; 9 Present Address: Department of Civil Engineering, University of Moratuwa, Moratuwa, Sri Lanka

**Keywords:** Climate-change impacts, Ocean sciences

## Abstract

Sandy coastlines adjacent to tidal inlets are highly dynamic and widespread landforms, where large changes are expected due to climatic and anthropogenic influences. To adequately assess these important changes, both oceanic (e.g., sea-level rise) and terrestrial (e.g., fluvial sediment supply) processes that govern the local sediment budget must be considered. Here, we present novel projections of shoreline change adjacent to 41 tidal inlets around the world, using a probabilistic, reduced complexity, system-based model that considers catchment-estuary-coastal systems in a holistic way. Under the RCP 8.5 scenario, retreat dominates (90% of cases) over the twenty-first century, with projections exceeding 100 m of retreat in two-thirds of cases. However, the remaining systems are projected to accrete under the same scenario, reflecting fluvial influence. This diverse range of response compared to earlier methods implies that erosion hazards at inlet-interrupted coasts have been inadequately characterised to date. The methods used here need to be applied widely to support evidence-based coastal adaptation.

## Introduction

The Low Elevation Coastal Zone (LECZ), defined globally as the areas within 10 m of mean sea-level^1^, is home to approximately 600 million people today, a number that is expected to approach one billion by 2050^2^. LECZ are heavily utilised for a wide range of activities including navigation, tourism, agriculture, marine/ecosystem resources and services, waste disposal, and recreational activities^3–8^. Some of the most heavily utilised LECZ areas are fronted by open sandy coasts, which comprise about one-third of the world's coastline^9^. They are complex morphodynamic systems that are commonly expected to erode during the twenty-first century due to rising sea levels and reduced sediment supply^10,11^. A majority of these sandy coasts are interrupted by tidal inlets^12–18^—i.e., inlet-interrupted coasts^18,19^, forming an important sub-set of open sandy coasts.

Both oceanic (e.g., change in mean sea-level) and terrestrial (e.g., change in fluvial sediment supply) processes contribute to the long-term (50–100 year) evolution of inlet-interrupted coasts^18,20–22^. This means that climate-change impacts (e.g., changes in temperature and precipitation, sea-level rise) and anthropogenic activities (e.g., land-use change, anthropogenic sediment retention, especially by dams) are likely to strongly influence the future behaviour of inlet-interrupted coasts. Such changes will inevitably lead to severe socio-economic impacts and generate adaptation needs along these heavily populated and utilised coastal zones^11,19,23–25^. This study was therefore undertaken with the overarching aim of deriving hitherto lacking projections of how this important and globally widespread type of coasts may evolve over the twenty-first century, taking account of both oceanic and terrestrial influences.

The behavioural dependence of inlet-interrupted coasts on both oceanic and terrestrial processes poses a major complication for long-term modelling of inlet-interrupted coasts. To adequately resolve the governing physical processes requires a holistic representation of Catchment-Estuary-Coastal (CEC) systems in a model^26^. Another complicating factor is the significant uncertainties associated with the anthropogenic and climate-change impacts that affect the future evolution of inlet-interrupted coasts^26–29^. Consequently, in addition to the uncertainties associated with the model techniques employed (i.e., model uncertainties), projections of inlet-interrupted coastline changes will also inherit the uncertainties associated with the climate-related drivers (e.g., sea-level rise (SLR), storm conditions, precipitation) and anthropogenic activities considered (i.e., input uncertainties). Therefore, it is important to understand and quantify the uncertainties associated with coastline change projections, which requires probabilistic as opposed to deterministic approaches. In view of these considerations, most existing coastal impact approaches are inadequate for supporting long-term planning and management of inlet-interrupted coasts, reflecting their: (1) partial rather than holistic consideration of sediment budgets in CEC system behaviour^20,22,26^, and (2) inability to provide long-term projections (particularly probabilistic projections) due to high computational demands and inherent model limitations (e.g., positive morphodynamic feedback loops)^26,29,30^. The Generalised-Scale-aggregated Model for Inlet-interrupted Coasts (G-SMIC)) used here overcomes these shortcomings and provides probabilistic assessments of the long-term evolution of inlet-interrupted coasts. To keep the problem tractable, the G-SMIC applications undertaken here account only for input uncertainties (i.e., uncertainties associated with climate change and anthropogenic activities) and do not account for model uncertainties.

## Computational approach

The computational approaches used in this study are fully described in the “Methods” section, and only a very brief synopsis is provided here. G-SMIC^22^, which is a substantial extension of the SMIC^18^, computes long-term shoreline change on inlet-interrupted coasts through the superposition of two main components: (1) shoreline change due to variations in sediment volume exchange between an estuary and the adjacent inlet-interrupted coast (${\mathrm{\Delta }V}_{\mathrm{T}}$), and (2) sea-level rise-induced landward movement of the shoreline (i.e., the Bruun effect).

The variation of sediment volume exchange between an estuary system and the adjacent inlet-interrupted coast (${\mathrm{\Delta }V}_{\mathrm{T}}$) is calculated as a summation of three governing processes^18^.

1 $$\Delta V_{{\text{T}}} = \Delta V_{{{\text{BI}}}} + \Delta V_{{{\text{BV}}}} + \Delta V_{{{\text{FS}}}}$$

where ${\Delta V}_{\mathrm{T}}$ is the cumulative change in the total sediment-volume exchange between the estuary and its adjacent coast, ${\Delta V}_{\mathrm{B}\mathrm{I}}$ is the sea-level rise-driven change in basin volume, ${\Delta V}_{\mathrm{B}\mathrm{V}}$ is the change in basin infill sediment volume due to variation in river discharge, and ${\Delta V}_{\mathrm{F}\mathrm{S}}$ is the change in fluvial sediment supply due to combined effects of climate change and anthropogenic activities (all volumes in ${\mathrm{m}}^{3}$).

G-SMIC requires four stochastic model inputs to compute the sediment volume exchange (${\Delta V}_{\mathrm{T}}$) between an estuary and its adjacent inlet-interrupted coast (viz., annual mean temperature (*T*), annual cumulative runoff (*Q*), change in global mean sea-level (${\Delta SL}_{\mathrm{G}}$), and human-induced erosion factor (${E}_{\mathrm{h}}$)). These stochastic inputs and other model inputs are used within a Monte-Carlo simulation to probabilistically determine the change in total sediment volume exchange (${\Delta V}_{\mathrm{T}}$) between CEC systems and adjacent inlet-interrupted coasts. To derive probabilistic outputs, the 10th, 50th, and 90th percentiles of the computed annual ${\Delta V}_{\mathrm{T}}$ are used to determine consequent shoreline change along the inlet-interrupted coasts. The effect of this total sediment volume change is assumed to shift the entire active coastal profile along the inlet-affected coast. This computed shoreline change is then combined with shoreline retreat due to the Bruun effect to obtain the total change in shoreline position directly related to changes in sea level, precipitation, and anthropogenic activities. Here, the extent of shoreline retreat due to the Bruun effect is determined using the modified Bruun rule presented by Vousdoukas et al. (2020)^10^.

Pilot applications of G-SMIC at three representative CEC systems (i.e., Alsea estuary, USA, Dyfi estuary, UK, and Kalutara estuary, Sri Lanka)^21,22^ demonstrated that model hindcasted rates of shoreline change over the 1985–2005 period compare well with the observed rates of shoreline changes presented by Luijendijk et al. (2018)^9^ at those locations. In addition to this, shoreline hindcasts were undertaken at eight more CEC systems, covering the full range of estuary surface areas considered in this study. The model hindcasts compare well with observed shoreline change rates presented by Luijendijk et al. (2018)^9^, with an average deviation of ~ 16% (see Supplementary Information Table S1). This enhanced validation of the model and provided confidence for wider applications of G-SMIC at other CEC systems around the world as done here.

Here, we apply G-SMIC at 41 CEC systems located in different climatic regions and geomorphic settings across the world to obtain shoreline change projections (including associated uncertainties) over the twenty-first century under the four IPCC Representative Concentration Pathways (viz., RCP 2.6, RCP 4.5, RCP 6.0, and RCP 8.5)^31^.

## Study locations

**Figure 1:**
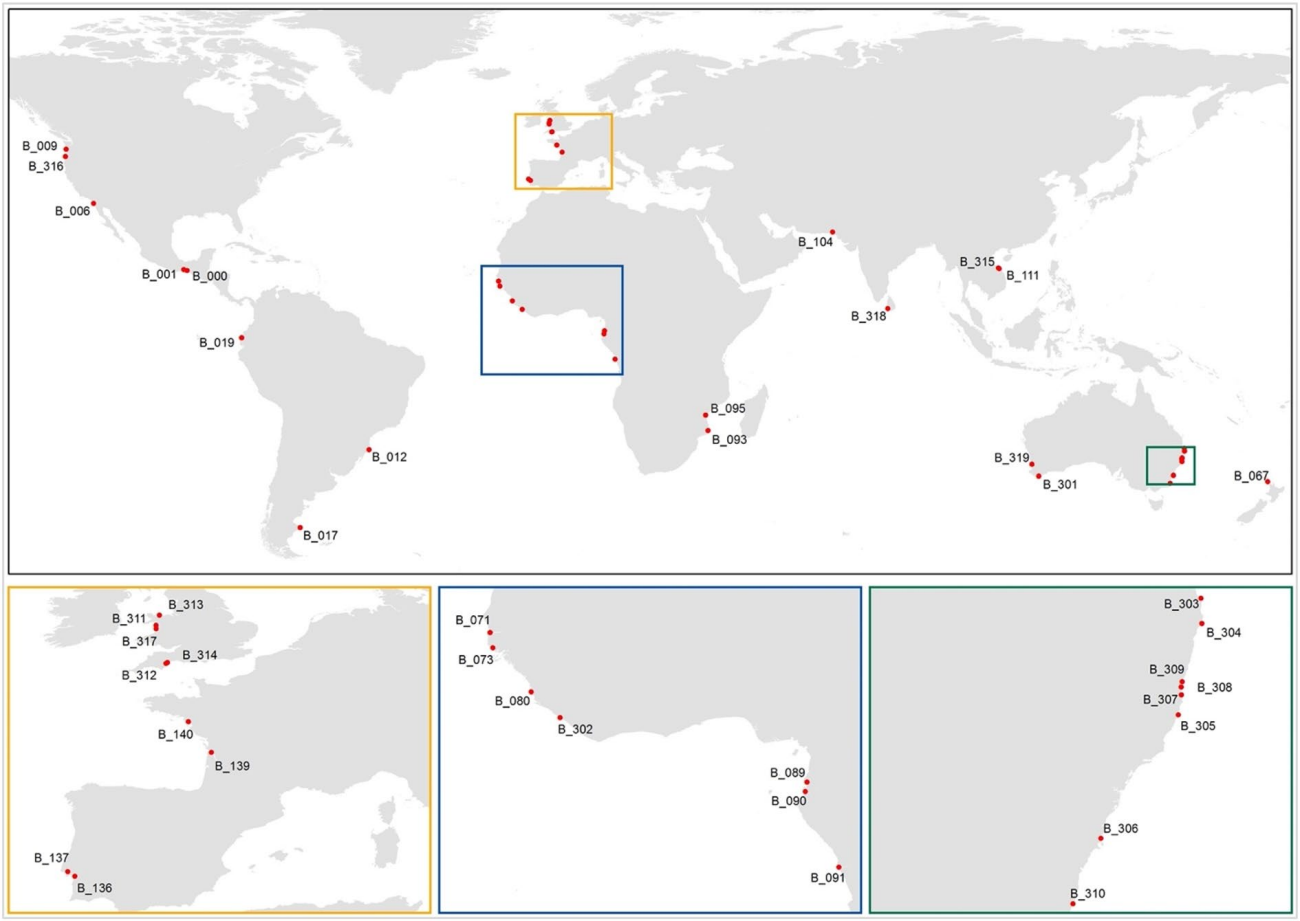
Locations of the selected catchment-estuary-coastal systems. Original data sources comprise: systems B_303 to B_310 are from the New South Wales (Australia) estuary dataset^33^; systems B_311 to B_314 are from the UK estuary dataset^32^; systems B_301, B_319 and B_315 are from the SMIC model application^18^; system B_302 is from the published literature^34,35^; systems B_316 to B_318 are from the original G-SMIC application^22^; all other systems are from the DIVA dataset^11^. Insert boxes and subplots below the main figure expand on areas that contain several closely-located systems which are not discernible in the main figure. Map is created using ArcGIS 10.7.1 (https://www.arcgis.com/).

The 41 CEC systems used in this study were selected based on data availability. These comprise 22 systems from the DIVA (Dynamic and Interactive Vulnerability Assessment) database^11^, 4 systems from the enhanced UK estuary database^32^, 8 systems from the New South Wales (Australia) estuary database^33^, 3 systems from the original G-SMIC application^22^, 3 systems from the previous SMIC application^18^, and one system in Liberia^34,35^. The location of the selected CEC systems, which cover North America, South and Central America, Europe, Africa, Asia, and Australasia, are shown in Fig. 1. The names, locations, and key system attributes that feed into G-SMIC are given in Supplementary Table S2.

## Sediment exchange volume projections

**Figure 2:**
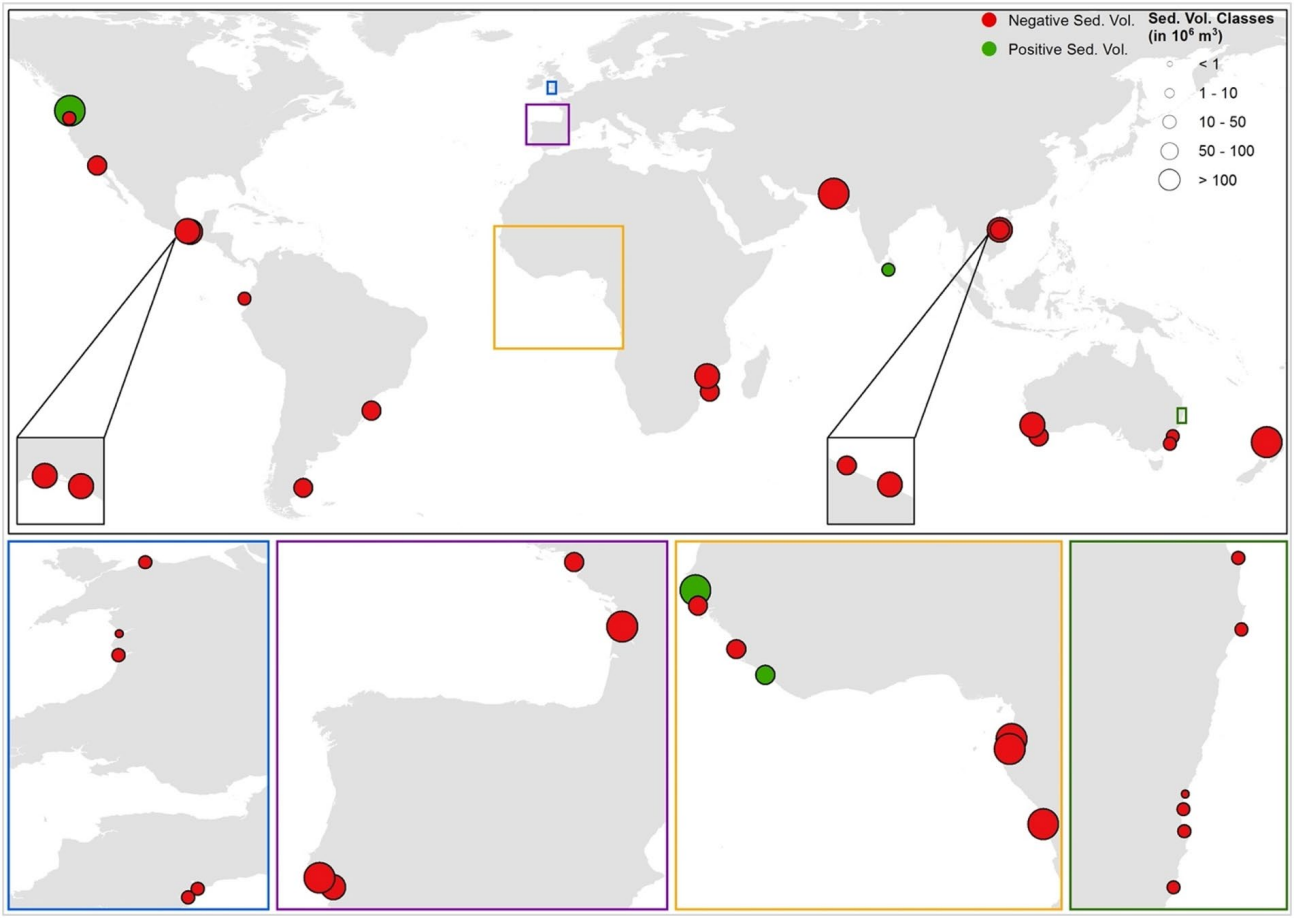
The projected change in the time-averaged 50th percentile values of the total sediment volume exchange (${\Delta V}_{T}$) between the estuary system and the adjacent coast at 41 catchment-estuary-coastal systems around the world, over the 2091–2100 period under RCP 8.5. ﻿ Negative and positive values of ${\Delta V}_{T}$ indicate sediment imported to the estuary from the adjacent coast and sediment exported from the estuary to the adjacent coast, respectively. Insert boxes and subplots below the main figure expand on areas that contain several closely-located systems which are not discernible in the main figure. Map is created using ArcGIS 10.7.1 (https://www.arcgis.com/).

The projected change in total sediment volume exchange (${\Delta V}_{\mathrm{T}}$) between the estuary-inlet systems and adjacent coasts at all 41 sites over 2091–2100 under RCP 8.5 is shown in Fig. 2. Of the fully probabilistic model results, the time-averaged 50th percentile values of ${\Delta V}_{\mathrm{T}}$ for the end-century period (2091–2100) are extracted and shown in Fig. 2. To provide insights into the uncertainty associated with these median projections, the time-averaged (2091–2100) 10^th^, 50^th^, and 90^th^ percentile values of ${\Delta V}_{\mathrm{T}}$ over the same period are extracted from the full CDFs and presented in Table S3. Mid-century (i.e., 2056–2065) projections of ${\Delta V}_{\mathrm{T}}$ are presented in Table S4.

These results show that 90% of the considered systems will be importing sediment by mid-century, under all RCPs, and will remain as importing systems by the end-century period (unless systems undergo major changes, such as dam removal). The magnitudes of ${\Delta V}_{\mathrm{T}}$ at all these sediment importing systems are projected to increase from mid- to end-century at all systems, with increases (in median projections) between 15 and 115% and 15–194% under RCP 2.6 and RCP 8.5, respectively. The two largest sediment import magnitudes (for all RCPs) are projected at the Muni (in Equatorial Guinea; median projections of -310, -398, -407, -573 Million Cubic Meter (MCM) by 2091–2100 under RCP 2.6, 4.5, 6.0, and 8.5, respectively) and Gabon systems (in Gabon; median projections of -253, -324, -332, and -467 MCM by 2091–2100 under RCP 2.6, 4.5, 6.0, and 8.5, respectively). Both these systems have extensive estuary surface areas, and thus, ${\Delta V}_{\mathrm{T}}$ at these systems is governed by the sediment demand resulting from the basin infilling (BI) process (see Table 1). Apart from these two systems, there are only four other importing systems where the projected median sediment import volumes exceed 100 MCM for all RCPs by the end century period (Kaipara, New Zealand; Zaire, DRC; Miani Hor, Pakistan; and Gironde, France). Among these four systems, Kaipara is the only system where ${\Delta V}_{\mathrm{T}}$ is governed by the basin infilling volume (BI), while the other three are influenced by fluvial sediment supply (FS) as well (see Table 1). At several of the importing systems, both basin infilling (BI) and fluvial sediment supply (FS) exert almost equal but opposite influences to the change in total sediment volume exchange (${\Delta V}_{\mathrm{T}}$) over the end-century period (e.g., Tubarao Lagoon, Brazil and Rio Chone, Ecuador). These inlet-estuary systems are attached to relatively large river catchments. Hence, the estuarine sediment demand due to basin infilling is reduced by the sizeable future increase in fluvial sediment supply from the catchment.

**Table 1:** Comparison of the primary sediment-supply control indicator (PSCI) and the dominant physical processes contributing to the total sediment volume exchange (${\Delta V}_{T}$) at the 41 catchment-estuary-coastal systems considered.

Label	System name	Estuary surface area (km^2^)	Catchment area (km^2^)	Primary sediment-supply control indicator (PSCI)	Dominant processes contributing to $\Delta {V}_{\mathrm{T}}$		
					BI	BV	FS
B_000	Mar Muerto	247	1448	0.17058	X		
B_067	Kaipara	780	5392	0.14466	X		
B_090	Gabon	750	5925	0.12658	X		
B_001	Lugana Superior	220	2028	0.10848	X		
B_006	San Diego	107	1167	0.09169	X		
B_111	Hue	100	1783	0.05609	X		
B_093	Inhambane	110	2377	0.04628	X		
B_137	Lisboa	320	8022	0.03989	X		
B_315	Thuan An	110	3800	0.02895	X		X
B_104	Miani Hor	314	11,066	0.02838	X		X
B_317	Dyfi	17.3	670	0.02582	X		
B_089	Muni	185	7995	0.02314	X		
B_073	Cacheu	130	5797	0.02243	X		
B_303	Tweed River	22.7	1066	0.02129	X		
B_012	Tubarao Lagoon	120	5636	0.02129	X		X
B_301	Wilson	48	2263	0.02121	X		
B_080	Freetown	206	11,110	0.01854	X		X
B_136	Setubal	110	6516	0.01688	X		
B_019	Rio Chone	32	2311	0.01385	X		X
B_312	Exe	19.2	1402	0.01368	X		
B_091	Zaire	520	40,989	0.01269	X		X
B_311	Mawddach	3.65	314	0.01160	X		
B_308	Nambucca River	12.6	1090	0.01156	X		X
B_313	Conwy	5.6	502	0.01107	X		
B_305	Hastings River	30	3594	0.00835	X		X
B_314	Teign	4.04	487	0.00830	X		X
B_316	Alsea	9.1	1225	0.00743	X		X
B_309	Bellinger River	8.2	1152	0.00712	X		X
B_139	Gironde	480	79,750	0.00602	X		X
B_304	Richmond River	38.4	6924	0.00555	X		X
B_095	Beira	130	23,830	0.00546	X		X
B_306	Shoalhaven	31.9	7087	0.00450	X		
B_307	Macleay River	31.6	11,347	0.00278	X		X
B_017	Rio Deseado	90	38,743	0.00232	X		
B_310	Bega River	3.8	1870	0.00203	X		
B_071	Gambia	120	70,018	0.00171	X		X
B_318	Kalutara	1.75	2778	0.00063			X
B_140	Loire	55	103,552	0.00053	X		X
B_009	Columbia River	340	669,403	0.00051			X
B_319	Swan	52	121,000	0.00043	**X**	**X**	
B_302	St Paul	1.02	20,281	0.00005			**X**

PSCI is the ratio between the estuary surface area and the river catchment area. Dominant contributing processes to the variation of ${\Delta V}_{T}$ are identified by examining the variations of the projected 50th﻿ percentile values of projected ${\Delta V}_{T}$ and the contributing processes (i.e., basin infilling (BI), basin volume change (BV), and fluvial sediment supply (FS)) over the 2020–2100 period (See Figure S1 and S2 of the Supplementary Information).

Of the 41 CEC systems considered here, only four systems are projected to export sediment (both at mid- and end-century periods and under all RCPs). These are Columbia River (USA), Gambia River (Senegal), St. Paul (Liberia), and Kalutara (Sri Lanka). A closer inspection of the relative contributions of the three governing processes considered in G-SMIC (Eq. 2), shows that, among these four systems, only the Gambia River system is governed by both fluvial sediment (FS) and basin infilling (BI) sediment components (see Table 1). The projected change in total sediment volume exchange (${\Delta V}_{\mathrm{T}}$) at the other three sediment exporting systems is governed by the fluvial sediment supply (FS) component (see Table 1). The Columbia River system is projected to export the largest amounts of sediment to the adjacent inlet-interrupted coast (median projections of 160, 321, 372, and 628 MCM by 2091–2100 under RCP 2.6, 4.5, 6.0, and 8.5, respectively). The projected increments in temperature and river discharge are the main reasons for such a large increase in fluvial sediment supply from the Columbia River. However, it should be noted that the effects of anthropogenic sediment retention along the Columbia River are not considered in this study, and it is plausible that some sediment retention structures will be removed before 2100^36,37^, increasing fluvial sediment supply to the coast.

We further analyse the relative contributions of basin infilling and fluvial sediment supply to the projected ${\Delta V}_{\mathrm{T}}$ to explore the possible existence of a threshold over which the fluvial sediment supply might have a dominant influence on the total change in sediment volume exchange (${\Delta V}_{\mathrm{T}}$). Such an a priori knowledge of the dominant governing process(s) could help streamlining detailed modelling efforts at CEC systems. In this analysis, the estuary surface area and the river catchment area are used as proxies for basin infilling volume and fluvial sediment supply, respectively. Here, the simple ratio between the estuary surface area and the river catchment area is considered as an indicator (hereon referred to as the Primary Sediment-supply Control Indicator (PSCI)), to investigate the relative contributions of basin infilling and fluvial sediment supply to the projected change in total sediment volume exchange (${\Delta V}_{\mathrm{T}}$).

The projected 50﻿th percentile values of ${\Delta V}_{\mathrm{T}}$ and the three physical processes contributing to ${\Delta V}_{\mathrm{T}}$ at the 41 CEC systems over the twenty-first century are examined in relation to the PSCI. As an example, Figure S1 and S2 illustrate the temporal evolution of these relative contributions under all RCPs for eight selected systems. A comparison of the projected changes in ${\Delta V}_{\mathrm{T}}$ and the three contributing processes for the CEC systems shown in Figure S1 and S2 show that Basin Infilling (BI) will have a dominant influence at all systems other than the St. Paul system, at which Fluvial sediment supply (FS) will be dominant. At Beira, Gironde and Alsea systems, Fluvial sediment supply (FS) also has a secondary but important influence on ${\Delta V}_{\mathrm{T}}$. The dominant processes, thus identified at all 41 CEC systems, were then compared with the corresponding PSCI values (Table 1). This analysis reveals that if PSCI greater than 0.01, the variation of projected ${\Delta V}_{\mathrm{T}}$ is likely to be governed by basin infilling (${\Delta V}_{\mathrm{B}\mathrm{I}}$) (Figure S2 and Table 1). When PSCI is less than 0.01 (at about 40% of the systems considered), the contribution of fluvial sediment supply to the variation in ${\Delta V}_{\mathrm{T}}$ is likely to be significant (i.e., cannot be neglected; Figure S1 and Table 1). Since shoreline change at tidal inlets is directly affected by the change in total sediment volume exchange (${\Delta V}_{\mathrm{T}}$) with the inlet-estuary systems, we postulate that PSCI can be used as a first-pass indicator of key physical processes that may affect the long-term evolution of inlet-interrupted coasts.

## Shoreline position change projections

**Figure 3:**
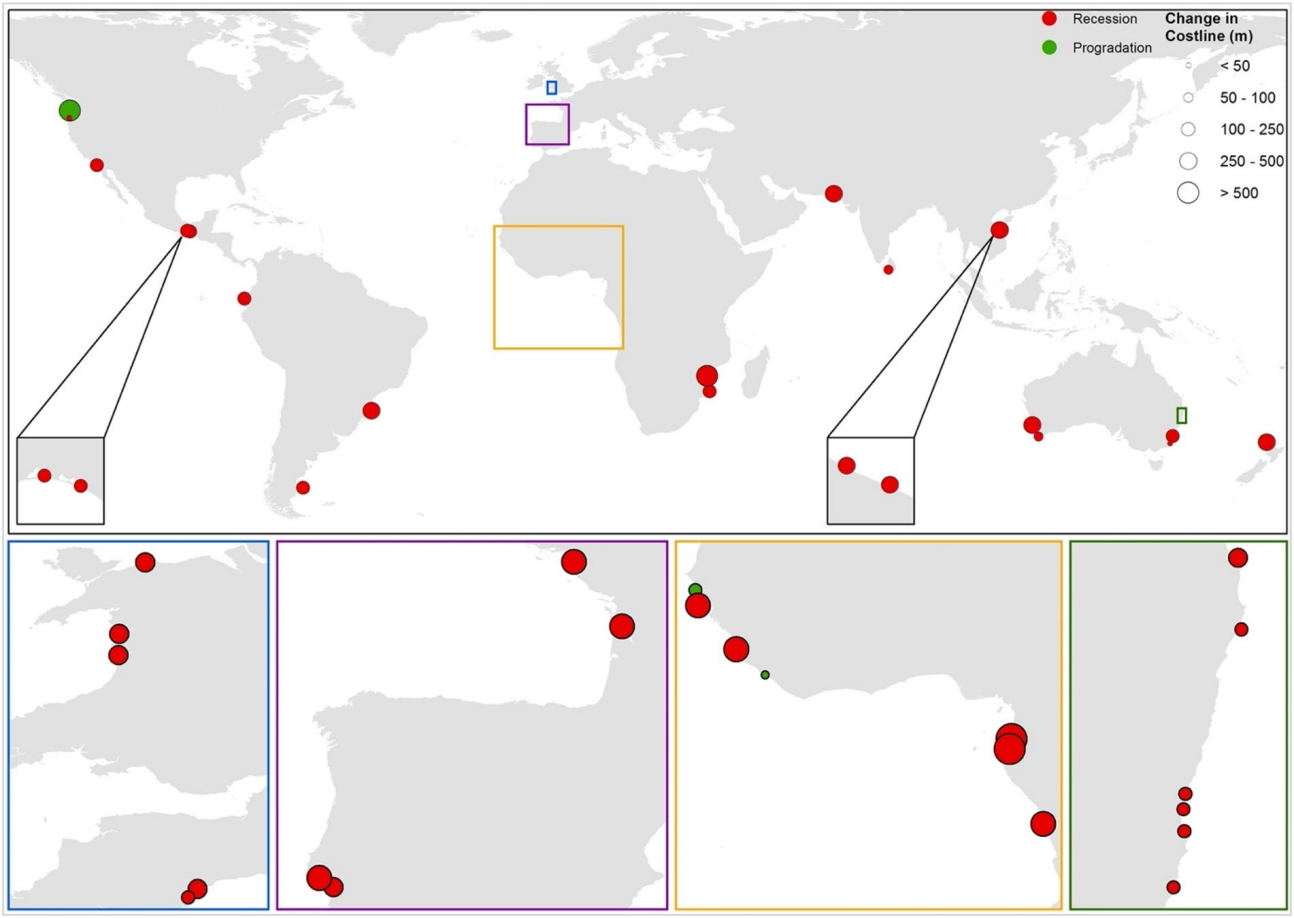
Projected shoreline position change at 41 catchment-estuary-coastal systems around the world by 2100 (relative to present-day) under RCP 8.5. The projected changes shown are associated with time-averaged (2091–2100) values of the 50th percentiles of the total sediment volume exchange (${\Delta V}_{T}$) between the estuary system and the adjacent coast plus the shoreline retreat due to the Bruun effect by 2091–2100. Insert boxes and subplots below the main figure expand on areas that contain several closely-located systems which are not discernible in the main figure. Map is created using ArcGIS 10.7.1 (https://www.arcgis.com/).

The projected shoreline position changes along the coastlines adjacent to the 41 CEC systems by 2091–2100, relative to present-day, are shown in Fig. 3 (for RCP 8.5). Shoreline position changes presented in Fig. 3 are calculated by combining shoreline changes due to ${\Delta V}_{\mathrm{T}}$ and the shoreline retreat due to the Bruun effect over the same period. The projected 10th, 50th, and 90th percentiles of shoreline position changes under all 4 RCPs by 2091–2100 (relative to present-day) computed from the probabilistic model outputs are presented in Table S5. Mid-century (2056–2065) projections of shoreline position change are presented in Table S6.

G-SMIC derived median projections of shoreline position change indicate that the combined effect of climate change (sea-level rise, changes in temperature and precipitation) and anthropogenic influences will induce shorelines adjacent to 90% of the systems to retreat over the twenty-first century under any RCP, with shorelines at 46% (RCP 2.6) and 68% (RCP 8.5) of the systems retreating more than 100 m by the end of the century.

Median projections of shoreline retreat across all sites projected to erode range from 6 to 632 m across the 4 RCPs. However, it should be noted that there are large uncertainties associated with these projections, with the largest and smallest ranges of projected shoreline position change for an individual site being 116 m and 2 m, respectively (across all RCPs). The largest range (10th﻿–90th﻿ percentile) of shoreline position change by the end-century period is projected at the Muni system for all RCPs (82 m and 116 m for RCP 2.6 and RCP 8.5, respectively). The minimum range of around 2 m is projected at Rio Chone, Bega River, Gambia, and Tweed River CEC systems for RCP 2.6, 4.5, 6.0, and 8.5, respectively. The large ranges in projections (Table S5) emphasise the need for a probabilistic approach to assess the long-term evolution of inlet-interrupted coasts in a way that can adequately inform adaptation strategies.

The difference between projections for mid- and end-century is only in the magnitude of shoreline retreat for 39 of the 41 CEC systems with projected increases (median values between the two time periods) ranging between 44 and102% and 58 and 157% across the systems under RCP 2.6 and RCP 8.5, respectively. At the two remaining CEC systems (Gambia and St Paul), shoreline retreat is projected for the mid-century period (relative to present-day), while shoreline progradation is projected for the end-century period (relative to present-day).

We compare the median shoreline position changes projected at the 41 CEC systems for 2091–2100 in this study with the findings of a recent study by Vousdoukas et al. (2020)^10^ that presented global projections of shoreline retreat/progradation over the twenty-first century under RCP 4.5 and RCP 8.5 (Table S7). Vousdoukas et al. (2020) 's projections depart from that of a straightforward application of the Bruun rule, in that their projections are based on a novel probabilistic approach that combines ambient shoreline change rates (based on satellite image-derived estimates over more than 30 years) and SLR driven future shoreline retreats computed via the modified Bruun rule (Eq. 8 in “Methods”). The comparison of the two sets of model results at eroding coasts (as projected by G-SMIC) indicates that the median shoreline retreats projected by G-SMIC are mostly larger than those presented by Vousdoukas et al. (2020)^10^ for the same locations. These differences are likely because Vousdoukas et al. (2020)’s projections do not fully account for inlet influences, and how they may change in the future, that are addressed in G-SMIC. A fundamental difference between the two sets of projections occurs at the Columbia, Gambia, and St. Paul systems, where G-SMIC projects shoreline progradation, whereas Vousdoukas et al. (2020)^10^ project shoreline retreat. This is likely because G-SMIC considers fluvial sediment supply and future changes therein (i.e., the main driver of shoreline progradation), while the approach adopted by Vousdoukas et al. (2020)^10^ does not consider this component of the sediment budget which is important, especially at CEC systems.

**Figure 4:**
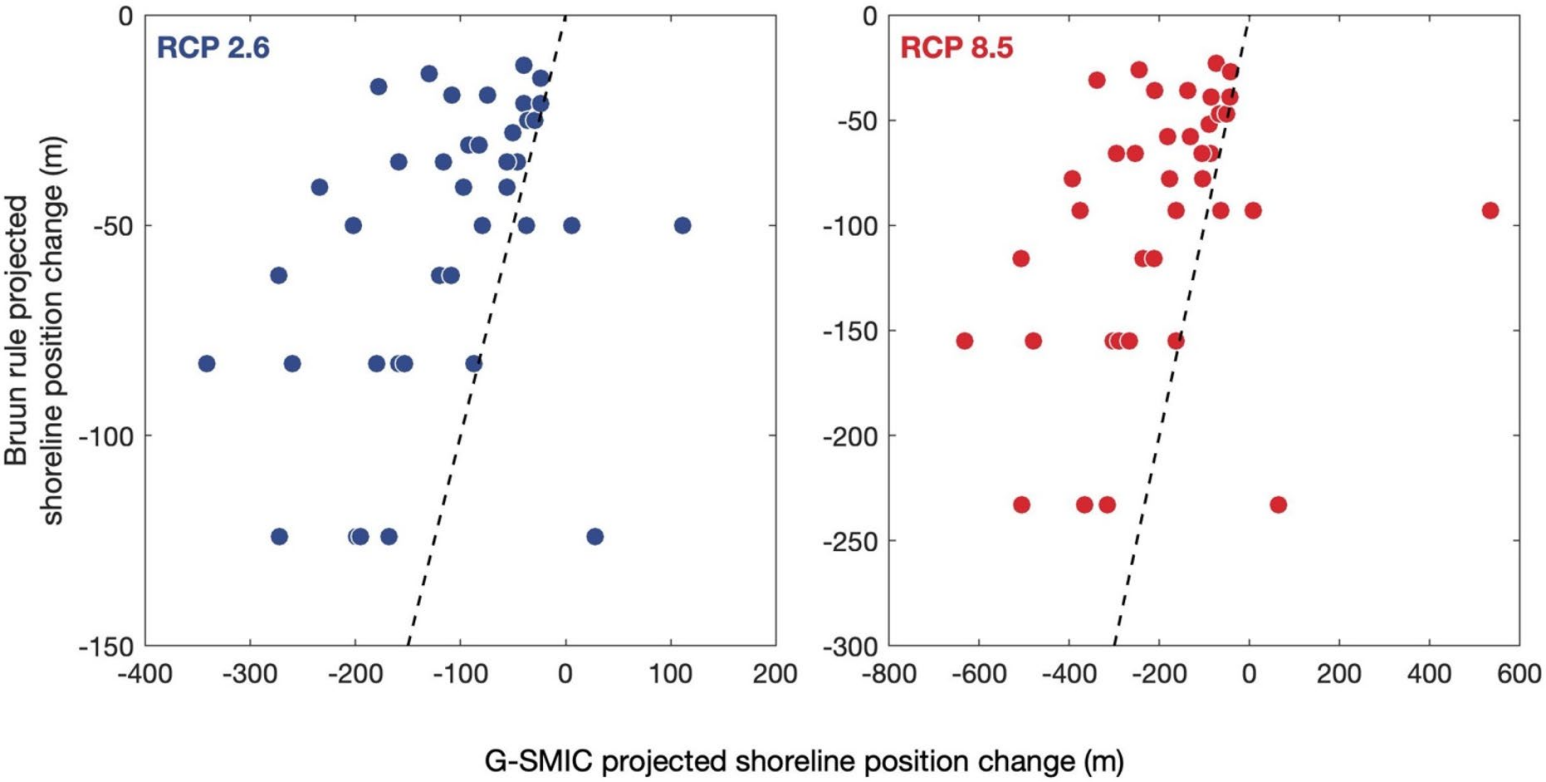
Comparison of projected median shoreline position change, derived through G-SMIC and the Bruun rule alone for 2091–2100. The diagonal dashed lines represent the perfect agreement between the two approaches.

Finally, G-SMIC projected median shoreline position change are compared with those derived only through the use of the Bruun rule alone (i.e., with Eq. 8), which is still routinely applied regardless of the presence of tidal inlets. This comparison (Table S8) shows that Bruun rule derived end-century median projections at about half of the CEC systems considered are more than 50% smaller than the corresponding projections obtained via G-SMIC, under all RCPs (see Fig. 4 for RCP 2.6 and RCP 8.5 median projection comparisons). This is in line with the original SMIC application to four small tidal inlets, where the Bruun effect was found to represent only 25–50% of the total projected shoreline change by 2100^18^. It is also noteworthy that three of the 41 CEC systems where G-SMIC projects shoreline progradation for the end-century period, the corresponding Bruun rule predictions indicate shoreline retreat. These discrepancies between projections re-iterate the inappropriateness of using the Bruun rule alone in the vicinity of tidal inlets and underline the importance of considering CEC systems in a holistic manner when deriving projections of future shoreline position change along inlet-interrupted coasts.

## Methods

### Model concept

#### Governing processes

**Figure 5:**
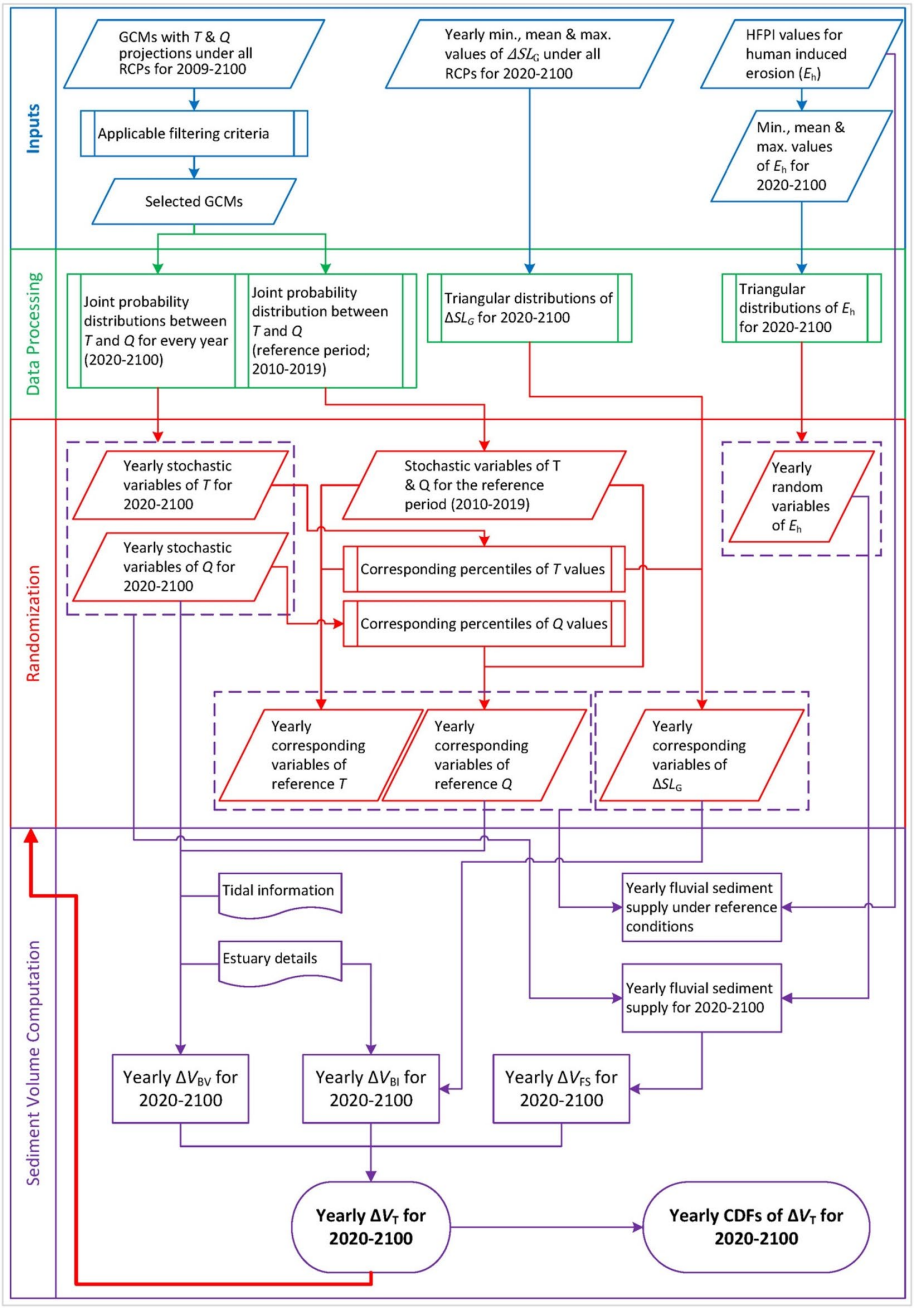
Schematic of the modelling approach adopted to probabilistically determine the change in total sediment volume exchange between an estuary system and the adjacent inlet-interrupted coast (Modified from Bamunawala et al. (2020)^22^).

The probabilistic modelling framework used in this study is described in detail by Bamunawala et al. (2020)^22^, and hence, only a summary is presented here. G-SMIC, which adopts the reduced-complexity modelling approaches first presented by Ranasinghe et al. (2013)^18^ for small tidal inlets, was substantially extended for general application at CEC systems by Bamunawala et al. (2020)^21^. In G-SMIC applications, long-term shoreline change at inlet-interrupted coasts is represented as the superposition of two main components: (1) shoreline change due to variation in sediment volume exchange between an estuary and its adjacent inlet-interrupted coast (${\Delta V}_{\mathrm{T}}$), and (2) sea-level rise-induced landward movement of the shoreline (i.e., the Bruun effect). The computational procedure of ${\Delta V}_{\mathrm{T}}$ is graphically shown in Fig. 5.

The variation of sediment volume exchange between an estuary system and its adjacent inlet-interrupted coast (${\Delta V}_{\mathrm{T}}$) is calculated as a summation of three processes^18^ as expressed in Eq. (1) , which is reproduced below for convenience.

$${\Delta V}_{\mathrm{T}}={\Delta V}_{\mathrm{B}\mathrm{I}}+{\Delta V}_{\mathrm{B}\mathrm{V}}+{\Delta V}_{\mathrm{F}\mathrm{S}}\quad \quad \quad(1)$$

where ${\Delta V}_{\mathrm{T}}$ is the cumulative change in the total sediment-volume exchange between the estuary and its adjacent coast, ${\Delta V}_{\mathrm{B}\mathrm{I}}$ is the sea-level rise-driven change in basin volume, ${\Delta V}_{\mathrm{B}\mathrm{V}}$ is the change in basin infill sediment volume due to variation in river discharge, and ${\Delta V}_{\mathrm{F}\mathrm{S}}$ is the change in fluvial sediment supply due to combined effects of climate change and anthropogenic activities (all volumes are in ${\mathrm{m}}^{3}$).

Accommodation space is the additional volume created within the basin due to a given increment in global sea level (${\Delta SL}_{\mathrm{G}}$ (m)). This volume will create a sediment demand in the estuary (${\Delta V}_{\mathrm{B}\mathrm{I}}$) that can be calculated as:

2 $$\Delta {V}_{\mathrm{B}\mathrm{I}}=-\mathrm{f}\mathrm{a}\mathrm{c}\left({A}_{\mathrm{b}}{\Delta SL}_{\mathrm{G}}\right)$$

where ${A}_{b}$ is estuary surface area (m^2^), and 'fac' (0 < fac < 1) is a factor that accounts for the morphological response lag. Following the SMIC^18^ and G-SMIC^21,22^ applications, this factor is taken as 0.5 in all the simulations. The negative sign in Eq. (2) denotes sediment demand from the inlet-estuary system due to sea-level rise.

Future changes in river flow may affect the ebb-flow volume of the tide. This ebb-flow volume variation will also change flow velocities within estuaries. An inlet-estuary system will undergo changes in its bed level and channel cross-section while striving to achieve its equilibrium flow conditions. This process involves a particular volume of sediment exchange between the inlet-estuary system and its adjacent inlet-interrupted coast (${\Delta V}_{\mathrm{B}\mathrm{V}}$), which can be calculated as:

3 $$\Delta {V}_{\mathrm{B}\mathrm{V}}=\frac{\Delta {Q}_{\mathrm{R}}{V}_{\mathrm{B}}}{\left(P+{Q}_{\mathrm{R}}\right)}$$

where ${Q}_{\mathrm{R}}$ is the present, annual averaged river flow into the basin during ebb, $\Delta {Q}_{\mathrm{R}}$ is the climate change-driven variation in river flow during ebb, ${V}_{\mathrm{B}}$ is the present basin volume, and *P* is the mean equilibrium ebb-tidal prism (all volumes in ${\mathrm{m}}^{3}$).

Projected changes in climate and anthropogenic activities will change fluvial sediment supply to the coasts^38–43^. This will have significant implications for the evolution of inlet-interrupted coasts. Previous studies^20,21,44,45^ have demonstrated that the empirical BQART model^46^ can be used to calculate the annual sediment throughput at the catchment scale.

4 $${Q}_{\mathrm{S}}=\mathrm{\omega }B{Q}^{0.31}{A}^{0.5}RT$$

where $\mathrm{\omega }$ is 0.02 or 0.0006 for the sediment volume (${Q}_{\mathrm{S}}$) expressed in kg/s or MT/year, respectively, *Q* is the annual river discharge from the catchment considered (${\mathrm{k}\mathrm{m}}^{3}/\mathrm{y}\mathrm{e}\mathrm{a}\mathrm{r}$), *A* is the catchment area (${\mathrm{k}\mathrm{m}}^{2}$), *R* is the relief of the catchment (km), and *T* ($>2^\circ{\rm C}$) is the catchment-wide mean annual temperature ($\mathrm{^\circ{\rm C} }$). Term '*B*' of the above equation represents the catchment sediment production and comprises glacial erosion (*I*), catchment lithology (*L*) that accounts for its soil type and erodibility, a reservoir trapping-efficiency factor (${T}_{\mathrm{E}}$), and human-induced erosion factor (${E}_{\mathrm{h}}$):

5 $$B=IL\left(1-{T}_{\mathrm{E}}\right){E}_{\mathrm{h}}$$

where glacial erosion (*I*) is expressed as:

6 $$I=1+\left(0.09{A}_{\mathrm{g}}\right)$$

where ${A}_{\mathrm{g}}$ is the ice cover percentage within the catchment area.

The BQART model uses nation-wide GDP/per capita and population density to approximate the human-induced erosion factor (${E}_{\mathrm{h}}$). Based on the global dataset used for the development of the BQART model, the optimum range of this factor (${E}_{\mathrm{h}}$) is suggested as 0.3–2.0^46^. However, Balthazar et al.^44^ demonstrate that this ${E}_{\mathrm{h}}$ factor can be better represented via high-resolution Human FootPrint Index (HFPI) data^47^. Following previous G-SMIC applications at three case studies^22^, HFPI data is used in this study to represent human-induced erosion factor (${E}_{\mathrm{h}}$) in river catchments. Due to the lack of a global database with all the reservoir attributes required to undertake a meaningful assessment of reservoir trapping efficiency, anthropogenic sediment retention was not considered in the model simulations.

#### Stochastic model inputs

Four stochastic model inputs are required by G-SMIC to compute the sediment volume exchange (${\Delta V}_{\mathrm{T}}$) between an estuary and its adjacent inlet-interrupted coast. These are annual mean temperature (*T*), annual cumulative runoff (*Q*), change in global mean sea-level (${\Delta SL}_{\mathrm{G}}$), and human-induced erosion factor (${E}_{\mathrm{h}}$). The sources from which these four stochastic model inputs and key CEC system properties were obtained for this study are summarised under CEC system data below. Additional properties of the selected CEC systems are presented in Table S2.

First, the stochastic model inputs are created for the study period considered (2020–2100). Here, per RCP, annual joint probability distributions are fitted between *T* and *Q* to represent their inter-dependencies^48,49^. Inputs for these distributions are obtained from the selected General Circulation Models (i.e., GCMs) (see under Model inputs associated with climate change and human activities). *T* and *Q* inputs are then stochastically generated from the fitted joint-probability distributions (100,000 values per variable, annually from 2020 to 2100). Annual stochastic model inputs of global mean sea-level change (${\Delta SL}_{\mathrm{G}}$) are generated from fitted triangular distributions per each RCP. Here, the annual triangular distributions of ${\Delta SL}_{\mathrm{G}}$ are fitted with the minimum, mean, and maximum projections of global mean sea-level change provided in the form of time series by Nicholls et al. (2014)^50^. As the main causes of sea-level rise^51^ (i.e., thermal expansion of oceans due to warming and increases in melting of glaciers and ice sheets) are directly related to increases in temperature, here, a direct relationship between *T* and ${\Delta SL}_{\mathrm{G}}$ is assumed for each RCP^52^. To achieve this direct relationship, percentiles of each annual mean temperature value are calculated. These percentiles and fitted triangular distributions of ${\Delta SL}_{\mathrm{G}}$ are used in conjunction to stochastically generate ${\Delta SL}_{\mathrm{G}}$ for 2020–2100 period, in a way that preserves the relationship between global warming and projected sea-level rise (100,000 values annually per each RCP). Fitted triangular distributions of human-induced erosion factor are used to generate stochastic model inputs of ${E}_{\mathrm{h}}$ for 2020–2100 period (100,000 random values per year). Following the method adopted in previous G-SMIC applications^22^, CEC system settings by 2019 are considered as the reference conditions. Climatic conditions over the last decade (i.e., 2010–2019) are used to obtain the reference *T* and *Q* values, to eliminate biased representation of the baseline climate that would likely have occurred if temperature and runoff values for only 2019 were used as the baseline.

#### Shoreline position

The above computed stochastic and other model inputs (Table S2) are then used within a Monte-Carlo simulation to probabilistically determine the change in total sediment volume exchange (${\Delta V}_{\mathrm{T}}$) between CEC systems and their adjacent inlet-interrupted coasts. To obtain probabilistic shoreline change projections, the 10th﻿, 50th﻿, and 90th﻿ percentiles of the computed annual ${\Delta V}_{\mathrm{T}}$ are used to determine the consequent shoreline changes along inlet-interrupted coasts. Here, ${\Delta V}_{\mathrm{T}}$ driven shoreline change is computed according to the simplified method used in SMIC applications^18^, where the change in total sediment volume exchange (${\Delta V}_{\mathrm{T}}$) is distributed uniformly along the potentially inlet-affected length of both up- and down-drift coasts. The effect of this total sediment volume change is assumed to shift the entire active coastal profile along the inlet-affected coast, expressed as:

7 $${dx}_{\mathrm{V}}=\frac{\Delta {V}_{\mathrm{T}}}{D{L}_{\mathrm{A}\mathrm{C}}}$$

where ${dx}_{\mathrm{V}}$ is coastline displacement (m) (in cross-shore direction) due to total sediment volume change, $\Delta {V}_{\mathrm{T}}$ is total sediment volume change (${\mathrm{m}}^{3}$), ${L}_{\mathrm{A}\mathrm{C}}$ is the total length of inlet-affected coastline (m), and *D* is the depth of closure (m). The extent of ${L}_{\mathrm{A}\mathrm{C}}$ in both up-drift and down-drift directions from a given inlet-estuary system is taken as the distance from the inlet to the next alongshore littoral barriers (e.g., headland, rock outcrop, tidal inlet, long groyne), or in the absence of such features, as 25 km, whichever is less, following Ranasinghe et al. (2013).

**Figure 6:**
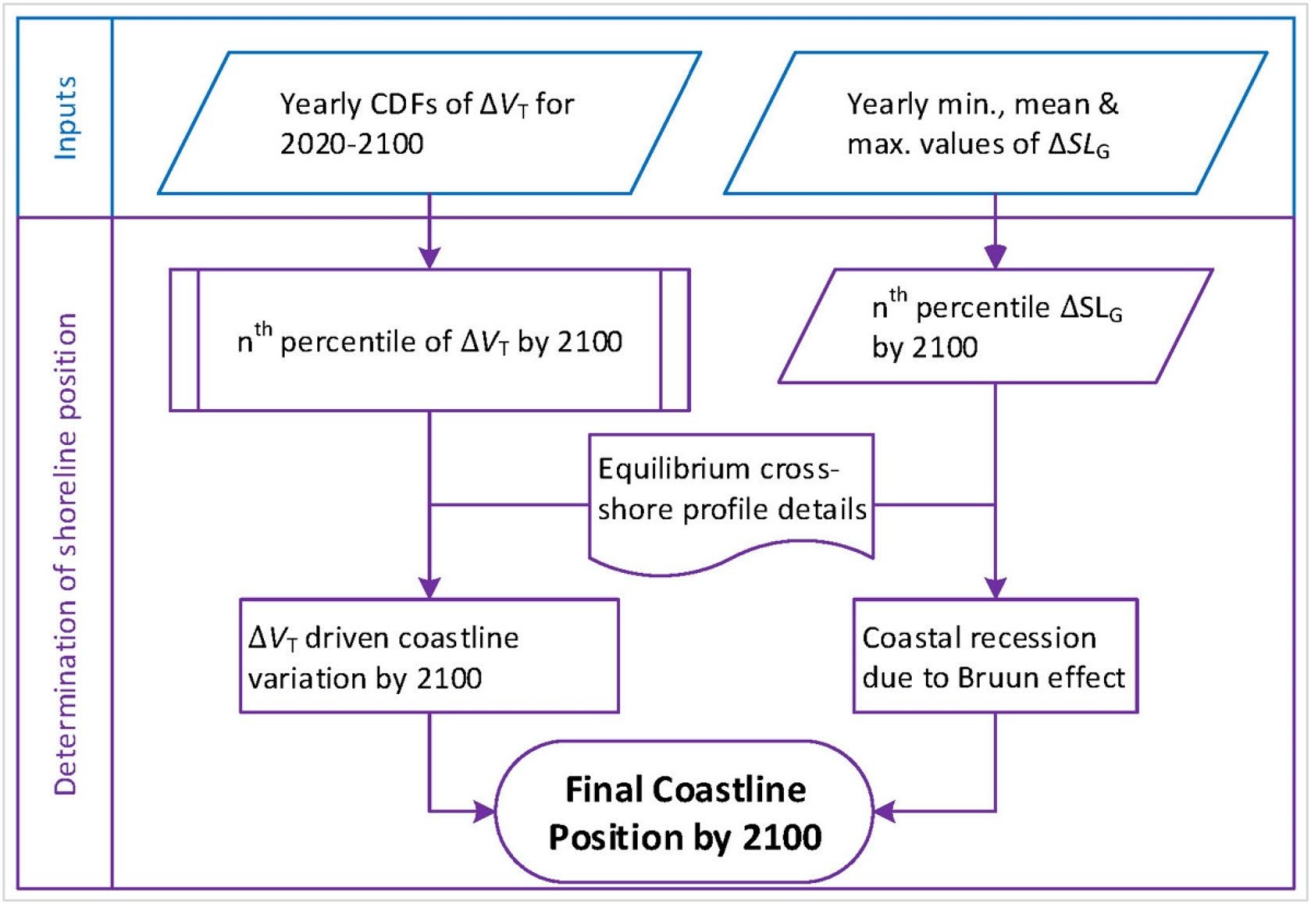
Modelling framework for determining the shoreline position change by 2100 (relative to present-day) along inlet-interrupted coastlines. Note: Annual empirical cumulative distributions (CDFs) are obtained from the sediment volume computations (${\Delta V}_{T}$) resulting from the modelling approach shown in Fig. 5.

Subsequently, the shoreline retreat due to the Bruun effect is superimposed on ${dx}_{\mathrm{V}}$ to obtain the final shoreline position change by 2100 relative to present-day conditions. The extent of shoreline retreat due to the Bruun effect is determined using the modified Bruun rule presented by Vousdoukas et al. (2020)^10^, expressed as:

8 $${dx}_{\mathrm{B}\mathrm{E}}=E\frac{1}{\mathrm{tan}\beta }SLR$$

where ${dx}_{\mathrm{B}\mathrm{E}}$ is the shoreline retreat (m) due to the Bruun effect, *E* is a correction factor for the Bruun effect that varies randomly within a fitted triangular distribution of minimum, maximum, and median values of 0.1, 1.0, and 0.75, respectively, $\mathrm{tan}\beta$ is the active profile slope^53,54^, and *SLR* is the projected sea-level rise. When considering long-term shoreline change, the active profile should ideally span from the base of the shoreface to the back of overwash deposition, if present^53,54^. However, due to the lack of local measurements at the CEC systems considered, the global estimates of active profile slopes presented by Athanasiou et al. (2019)^55^ are used in this study. An illustration of the method adopted to determine the shoreline position change by 2100 (relative to present-day) is presented in Figs. 5 and 6.

The change in shoreline position computed by G-SMIC assumes no scarcity of sediment supply from the coast or the catchment. However, this assumption may not hold in some situations: e.g., when a basin consists of a stiff clay bed, when the nearshore zone consists of non-erodible rock covered with a thin layer of sand, when large volumes of sand enter embayed beaches due to episodic headland bypassing^18^. In addition, G-SMIC projections assume that any sediment exported from an estuary is coarse enough to remain in the high-energy nearshore system. However, fluvial sediment loads in some systems include finer sediment, which can contribute to sediment budgets for estuaries but not shorelines. In sediment exporting systems that contain a high percentage of fine fluvial sediments, a proportion of the exported sediment volume may be lost to the sea. In such situations, the shoreline progradation (or the offsetting of the Bruun effect) computed by G-SMIC is likely to be an overestimate. Furthermore, the model application presented here assumes that there are no physical obstructions (e.g., seawalls, revetments, rocky cliffs) to shoreline retreat at the CEC systems considered, which is often not the case for developed coastlines. Such physical obstructions to shoreline retreat are present at about 60% of the CEC systems considered here. Given these assumptions, in essence, what G-SMIC computes is the “potential” shoreline position change. However, to improve readability, ‘shoreline position change’ is used throughout the manuscript when referring to ‘potential shoreline position change’.

It is important to note that the shoreline changes calculated in the G-SMIC model represent the change in shoreline position due to future variations in fluvial sediment supply, river discharge, and sea level^21,22^. Therefore, if it is desired to compute absolute shoreline positions in a future period, it is necessary to superimpose the reference rate of shoreline changes^9^, arising from the sediment balance during the reference period, to those obtained from G-SMIC applications. G-SMIC will only provide an absolute shoreline position in a future period when the projected rate of shoreline change is much larger than those under reference conditions.

### CEC system data

Connecting river catchment areas of the selected inlet-estuary systems are determined from the river network and basin information given by Lehner et al. (2008)^56^. These river basin extents are then used in conjunction with one arc-second resolution digital elevation model obtained from USGS Earth Explorer tool^57^ and Human FootPrint Index data^47^ to determine the catchment relief (*R*) and human-induced erosion factor (${E}_{\mathrm{h}}$) values, respectively. The annual average tidal amplitude and the annual number of tidal cycles at the CEC systems are obtained from the harmonic analysis of the TOPEX-POSEIDON global inverse solutions^58^. Catchment-averaged lithological factors (*L*) for the selected systems are obtained from the global map presented together with the BQART model development^46^. The depth of closure and the active coastal-profile slope values at the CEC systems are obtained from a published global dataset^55^. The estuary surface-area values presented in DIVA^11^, UK^32^, and NSW^33^ estuary datasets were checked against a published global estuary dataset^59,60^ to ensure the reliability of the estuary surface-area values used in the model applications, as this parameter affects two of the three components (i.e., basin infilling volume (${\Delta V}_{\mathrm{B}\mathrm{I}}$) and basin volume change (${\Delta V}_{\mathrm{B}\mathrm{V}}$)) of the total sediment volume exchange (${\Delta V}_{\mathrm{T}}$) calculations in the model. As the DIVA dataset does not contain basin volume information, the basin volume values required for sediment volume calculation are obtained through a fitted linear regression model that estimates basin-volume magnitude given estuary surface area and tidal amplitude values^61^. The input (i.e., basin volume, estuary surface area, and tidal amplitude) for the linear regression model development are obtained by combining three estuary databases from UK^32^, USA^62^, and New South Wales (Australia)^33^. The final combined dataset used for regression model development contained 324 estuary systems.

### Model inputs associated with climate change and human activities

Catchment wide mean annual temperature and annual cumulative runoff values for the period 2010–2100 are obtained from four GCMs (GFDL-CM3, GFDL-ESM2G, GFDL-ESM2M, and IPSL-CM5A-MR). The selection of the above four GCMs is based on the availability of temperature and runoff projections for all RCPs over the 2010–2100 interval, the spatial resolution of the GCM outputs (< 2.5 $^\circ$), and available regional GCM assessment literature (e.g., Climate change projections for the Australian territories^63^). Time series of minimum, median and maximum projections of global mean sea-level rise per RCP are obtained from Nicholls et al. (2014)^50^. Annual stochastic model inputs of human-induced erosion factor (${E}_{\mathrm{h}}$) are generated from fitted triangular distributions, in which linearly increasing minimum, median, and maximum values of ${E}_{\mathrm{h}}$ are assumed at each CEC system over the study period. Following the original G-SMIC application^22^, these increments by 2100 are assumed to be 10 (minimum), 15 (median), and 20 (maximum) percent of the reference condition. It should be noted that different methods of translating present-day HFPI into the future would result in diverse projections of fluvial sediment supply from the river catchments. A dedicated study investigating different socio-economic pathways, including changes in land use pattern, reservoir sediment trapping, and the introduction of flood control/water diversion structures, could provide more detailed representations of these increased human-induced impacts on the environment.

### Computational procedure

The above mentioned four stochastic model inputs (i.e., *T*, *Q*, ${\Delta SL}_{\mathrm{G}}$, and ${E}_{\mathrm{h}}$) are used in a Monte-Carlo framework (shown in Fig. 5) to obtain probabilistic projections of the change in total sediment volume exchange (${\Delta V}_{\mathrm{T}}$) at the 41 CEC systems. Flowchart of the modelling approach used to determine the shoreline position change is shown in Fig. 6.

All G-SMIC simulations undertaken here spanned the period 2020–2100, with 100,000 individual simulations per year, per RCP, within a Monte-Carlo framework. The entire modelling effort (for the 4 RCPs, per each CEC system) takes about 15 min on a standard 4-core laptop.

## Supplementary Information

Supplementary Information.
