# Supplementary material for: Twenty-first-century projections of shoreline change along inlet-interrupted coastlines
Source: Sci Rep. 2021 Jul 7;11:14038. doi: 10.1038/s41598-021-93221-9 (PMC8263749; doi:10.1038/s41598-021-93221-9)
Supplement: Supplementary file 1 — Supplementary Information. [file 41598_2021_93221_MOESM1_ESM.docx]

**SUPPLEMENTARY INFORMATION: 21^st^-century projections of shoreline change along inlet-interrupted coastlines**

Janaka Bamunawala^1,2*#^, Roshanka Ranasinghe^1,2,3^, Ali Dastgheib^2^, Robert J. Nicholls^4^, A. Brad Murray^5^, Patrick L. Barnard^6^, T. A. J. G. Sirisena^1^, Trang Minh Duong^1,2,3^, Suzanne J. M. H. Hulscher^1^, and Ad van der Spek^7,8^

^1^ Department of Water Engineering & Management, University of Twente, P.O. Box 217,

7500 AE Enschede, The Netherlands

^2^ IHE Delft Institute for Water Education, P.O. Box 3015, 2601 DA Delft, The Netherlands

^3^ Harbour, Coastal and Offshore Engineering, Deltares, P.O. Box 177, 2600 MH Delft, The Netherlands

^4^ Tyndall Centre for Climate Change Research, University of East Anglia, Norwich, NR4 7TJ, UK

^5^ Division of Earth and Ocean Sciences, Nicholas School of the Environment, Center for Nonlinear and Complex Systems, Duke University, Box 90229, Durham, NC 27708-0229 USA

^6^ United States Geological Survey, Pacific Coastal and Marine Science Center, 2885 Mission Street,

Santa Cruz, CA 95060, USA

^7^ Applied Morphodynamics, Deltares, P.O. Box 177, 2600 MH Delft, The Netherlands

^8^ Department of Physical Geography, Faculty of Geosciences, Utrecht University, P.O. Box 80115, 3508 TC Utrecht, The Netherlands

* Corresponding author: Janaka Bamunawala ([bamunawala@uom.lk](mailto:bamunawala@uom.lk))

^#^ Presently at Department of Civil Engineering, University of Moratuwa, Moratuwa, Sri Lanka

**Supplementary Figure (S1)**


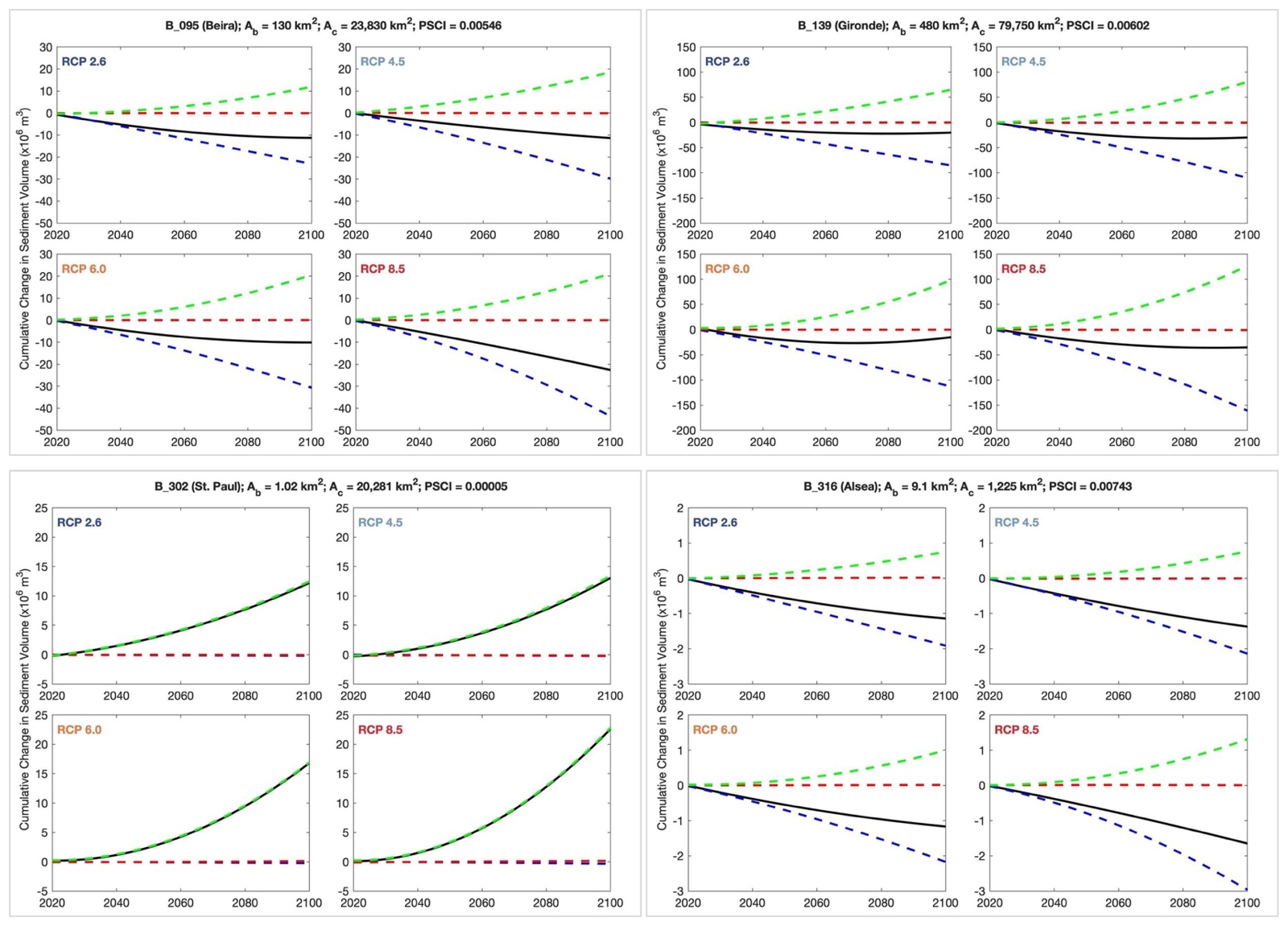


Figure S1: Projected variations of the 50^th^ percentile values of the change in total sediment volume exchange ($\boldsymbol{\Delta V}_{\boldsymbol{T}}$) and the different contributing processes at four different CEC systems where the Primary Sediment-supply Control Indicator (PSCI) is less than 0.01 for RCP 8.5 over the study period 2020-2100. The solid black line indicates the variation of $\boldsymbol{\Delta V}_{\boldsymbol{T}}$, while the dashed blue, red and green lines indicate the variations of basin infilling (BI), basin volume change (BV) and fluvial sediment supply (FS), respectively

**Supplementary Figure (S2)**


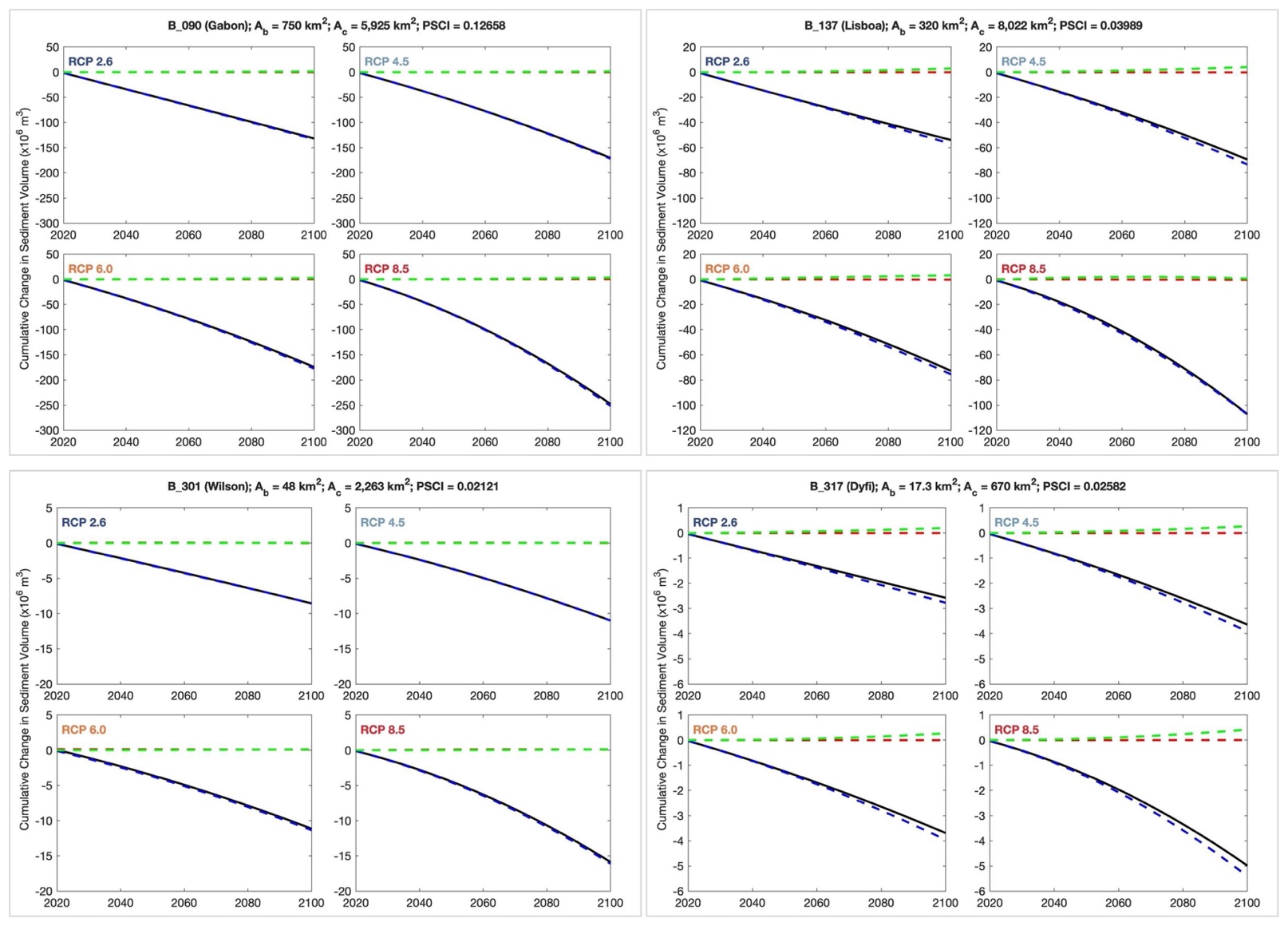


Figure S2: Projected variations of the 50^th^ percentile values of the change in total sediment volume exchange ($\boldsymbol{\Delta V}_{\boldsymbol{T}}$) and the different contributing processes at four different CEC systems where the Primary Sediment-supply Control Indicator (PSCI) is greater than 0.01 for RCP 8.5 over the study period 2020-2100. The solid black line indicates the variation of $\boldsymbol{\Delta V}_{\boldsymbol{T}}$, while the dashed blue, red and green lines indicate the variations of basin infilling (BI), basin volume change (BV) and fluvial sediment supply (FS), respectively

**Supplementary Table (S1)**

Table S1: Comparison of model hindcasted rates of shoreline position change over 1986-2005 with corresponding observed rates of shoreline change presented by Luijendijk et al. (2018)^14^. Positive and negative values indicate shoreline progradation and retreat, respectively.

| Label | CEC system name | Estuary surface area (km^2^) | River catchment area (km^2^) | Rate of coastline change over 1986-2005 (m/yr) | |
| --- | --- | --- | --- | --- | --- |
|  |  |  |  | Luijendijk et al. (2018)^14^ | G-SMIC |
| B_319 | Swan | 52 | 121,000 | -1.0 | -1.2 |
| B_071 | Gambia | 120 | 70,018 | 1.7 | 1.4 |
| B_089 | Muni | 185 | 7,995 | -1.3 | -1.5 |
| B_080 | Freetown | 206 | 11,110 | -2.4 | -2.9 |
| B_137 | Lisboa | 320 | 8,022 | -3.2 | -3.8 |
| B_139 | Gironde | 480 | 79,750 | -6.1 | -5.8 |
| B_091 | Zaire | 520 | 40,989 | -3.2 | -3.9 |
| B_090 | Gabon | 750 | 5,925 | -2.7 | -3.1 |

**Supplementary Table (S2)**

Table S2: Properties of the catchment-estuary-coastal systems considered in this study. Note: Estuary surface area values are available from the original datasets (i.e., global estuary dataset of the DIVA framework ^1^, The enhanced UK estuary database ^2^, The New South Wales estuary database ^3^, SMIC application dataset ^4^, Inlet-estuary systems considered in the original G-SMIC application ^5^. Information on the inlet-estuary system in Liberia is from published literature ^6,7^); Basin volume values are derived through the linear regression model developed ^8^ to project the basin volume magnitude based on the estuary surface area and the tidal amplitude; Lithology factors of the catchment areas are obtained from the global map presented with the BQART model development ^9^; Catchment relief values are calculated from the one arc-second resolution digital elevation models obtained from USGS earth explorer tool ^10^; Human-induced erosion factor is calculated from the Human FootPrint Index (HFPI) data ^11^; Depth of closure and active profile slope values are from the global dataset presented by Athanasiou et al. (2019)^12^

| Label | System name | Longitude (degrees East) | Latitude (degrees North) | Catchment area (km^2^) | Estuary area (km^2^) | Basin volume (MCM) | Lithology factor | Catchment relief (km) | Human-induced erosion factor | Depth of closure (m) | Total length of inlet-affected coast (km) | Active profile slope |
| --- | --- | --- | --- | --- | --- | --- | --- | --- | --- | --- | --- | --- |
| B_000 | Mar Muerto | -93.981 | 16.056 | 1,448 | 247 | 456.3 | 1.0 | 2.478 | 1.0219 | 8 | 50 | 0.013 |
| B_001 | Lugana Superior | -94.732 | 16.273 | 2,028 | 220 | 406.4 | 1.5 | 2.26 | 0.8538 | 8 | 50 | 0.004 |
| B_006 | San Diego | -117.139 | 32.659 | 1,167 | 107 | 197.7 | 1.5 | 1.75 | 1.1160 | 12 | 20 | 0.008 |
| B_009 | Columbia River | -123.974 | 46.238 | 669,403 | 340 | 628.1 | 0.75 | 4.18 | 0.5757 | 20 | 50 | 0.005 |
| B_012 | Tubarao Lagoon | -48.788 | -28.483 | 5,636 | 120 | 221.7 | 1.5 | 1.8 | 0.7990 | 13 | 10 | 0.007 |
| B_017 | Rio Deseado | -65.868 | -47.762 | 38,743 | 90 | 166.3 | 1.0 | 2.4 | 0.4821 | 15 | 30 | 0.005 |
| B_019 | Rio Chone | -80.406 | -0.629 | 2,311 | 32 | 59.1 | 1.5 | 0.67 | 0.9383 | 8 | 15 | 0.006 |
| B_067 | Kaipara | 174.158 | -36.407 | 5,392 | 780 | 1,441.1 | 2.0 | 0.77 | 0.8925 | 17 | 50 | 0.007 |
| B_071 | Gambia | -16.582 | 13.426 | 70,018 | 120 | 221.7 | 2.0 | 1.54 | 0.7335 | 19 | 20 | 0.002 |
| B_073 | Cacheu | -16.375 | 12.146 | 5,797 | 130 | 240.2 | 2.0 | 0.08 | 0.8172 | 19 | 25 | 0.002 |
| B_080 | Freetown | -13.198 | 8.515 | 11,110 | 206 | 380.6 | 1.5 | 0.98 | 0.8627 | 20 | 15 | 0.002 |
| B_089 | Muni | 9.646 | 1.053 | 7,995 | 185 | 341.8 | 0.5 | 1.15 | 0.5803 | 20 | 50 | 0.003 |
| B_090 | Gabon | 9.482 | 0.246 | 5,925 | 750 | 1385.6 | 0.5 | 0.92 | 0.4684 | 20 | 50 | 0.004 |
| B_091 | Zaire | 12.302 | -6.010 | 40,989 | 520 | 960.7 | 0.5 | 1.08 | 0.6847 | 15 | 50 | 0.005 |
| B_093 | Inhambane | 35.395 | -23.802 | 2,377 | 110 | 203.2 | 2.0 | 0.24 | 0.7744 | 11 | 30 | 0.013 |
| B_095 | Beira | 34.807 | -19.884 | 23,830 | 130 | 240.2 | 0.75 | 1.85 | 0.6543 | 9 | 25 | 0.002 |
| B_104 | Miani Hor | 66.329 | 25.565 | 11,066 | 314 | 580.1 | 2.0 | 2.33 | 0.7718 | 10 | 50 | 0.006 |
| B_111 | Hue | 107.647 | 16.564 | 1,783 | 100 | 122.0 | 1.0 | 1.45 | 0.8541 | 20 | 20 | 0.002 |
| B_136 | Setubal | -8.723 | 38.453 | 6,516 | 110 | 203.2 | 1.0 | 0.44 | 0.9200 | 14 | 25 | 0.018 |
| B_137 | Lisboa | -9.096 | 38.701 | 8,022 | 320 | 591.2 | 1.5 | 0.98 | 0.9504 | 15 | 35 | 0.015 |
| B_139 | Gironde | -0.930 | 45.491 | 79,750 | 480 | 886.8 | 1.0 | 3.19 | 0.9072 | 15 | 50 | 0.003 |
| B_140 | Loire | -2.226 | 47.231 | 103,552 | 55 | 101.6 | 1.0 | 1.86 | 0.9413 | 15 | 20 | 0.003 |
| B_301 | Wilson | 117.335 | -35.027 | 2,263 | 48 | 85.0 | 0.5 | 0.474 | 0.5570 | 19 | 15 | 0.02 |
| B_302 | St Paul | -10.801 | 6.378 | 20,281 | 1.02 | 1.9 | 0.5 | 1.649 | 0.7945 | 10 | 20 | 0.005 |
| B_303 | Tweed River | 153.558 | -28.169 | 1,066 | 22.7 | 41.9 | 1.0 | 1.171 | 0.8227 | 13 | 5 | 0.004 |
| B_304 | Richmond River | 153.591 | -28.877 | 6,924 | 38.4 | 70.9 | 1.0 | 1.152 | 0.6756 | 13 | 15 | 0.012 |
| B_305 | Hastings River | 152.920 | -31.425 | 3,594 | 30 | 55.4 | 1.0 | 1.245 | 0.7627 | 13 | 12 | 0.009 |
| B_306 | Shoalhaven River | 150.767 | -34.895 | 7,087 | 31.9 | 58.9 | 1.5 | 1.475 | 0.6265 | 13 | 10 | 0.008 |
| B_307 | Macleay River | 153.026 | -30.870 | 11,347 | 31.6 | 58.4 | 1.0 | 1.583 | 0.6076 | 14 | 10 | 0.01 |
| B_308 | Nambucca River | 153.016 | -30.654 | 1,090 | 12.6 | 23.3 | 1.0 | 1.08 | 0.7677 | 14 | 10 | 0.01 |
| B_309 | Bellinger River | 153.036 | -30.502 | 1,152 | 8.2 | 15.1 | 1.0 | 1.558 | 0.7283 | 14 | 12 | 0.01 |
| B_310 | Bega River | 149.985 | -36.704 | 1,870 | 3.8 | 7.0 | 0.5 | 1.3 | 0.6637 | 14 | 5 | 0.017 |
| B_311 | Mawddach | -4.064 | 52.710 | 314 | 3.6 | 6.7 | 0.5 | 0.878 | 0.9425 | 10 | 12 | 0.003 |
| B_312 | Exe | -3.417 | 50.608 | 1,402 | 19.2 | 35.4 | 0.5 | 0.517 | 1.0585 | 11 | 5 | 0.006 |
| B_313 | Conwy | -3.849 | 53.300 | 502 | 5.6 | 10.3 | 0.5 | 0.863 | 0.8714 | 8 | 7 | 0.006 |
| B_314 | Teign | -3.495 | 50.539 | 487 | 4.1 | 7.5 | 0.5 | 0.589 | 1.0471 | 11 | 5 | 0.007 |
| B_315 | Thuan An | 107.622 | 16.575 | 3,800 | 110 | 178.0 | 1.0 | 1.2 | 0.8200 | 20 | 10 | 0.003 |
| B_316 | Alsea | -124.089 | 44.422 | 1,225 | 9.1 | 20.0 | 1.0 | 1.25 | 0.6100 | 22 | 15 | 0.012 |
| B_317 | Dyfi | -4.069 | 52.534 | 670 | 17.3 | 45.0 | 0.75 | 0.66 | 0.9200 | 10 | 12 | 0.007 |
| B_318 | Kalutara | 79.956 | 6.576 | 2,778 | 1.75 | 5.3 | 0.5 | 2.25 | 0.9300 | 7 | 30 | 0.005 |
| B_319 | Swan | 115.734 | -32.055 | 121,000 | 52 | 312.0 | 0.5 | 0.8 | 0.5800 | 17 | 30 | 0.003 |

**Supplementary Table (S3)**

Table S3: The projected 10^th^, 50^th^, and 90^th^ percentiles of the time-averaged (2091-2100) change in total sediment volume exchange ($\boldsymbol{\Delta V}_{\boldsymbol{T}}$) at the 41 catchment-estuary-coastal systems under the 4 IPCC RCPs. Note: The time-averaged values of change in total sediment volume exchange were computed by averaging the projected 10^th^, 50^th^, and 90^th^ percentile values of $\boldsymbol{\Delta V}_{\boldsymbol{T}}$ over 2091-2100 period; negative signs denote sediment imported into the estuary

| Label | System Name | Time-averaged (2091-2100) values of the change in total sediment volume exchange ($\Delta V_{T}$) ($\times{10}^{6} m^{3}$) | | | | | | | | | | | |
| --- | --- | --- | --- | --- | --- | --- | --- | --- | --- | --- | --- | --- | --- |
|  |  | RCP 2.6 | | | RCP 4.5 | | | RCP 6.0 | | | RCP 8.5 | | |
|  |  | 10^th^ | 50^th^ | 90^th^ | 10^th^ | 50^th^ | 90^th^ | 10^th^ | 50^th^ | 90^th^ | 10^th^ | 50^th^ | 90^th^ |
| B_000 | Mar Muerto | -45.5 | -35.5 | -26.3 | -57.6 | -47.3 | -37.9 | -58.3 | -47.8 | -38.0 | -83.4 | -69.5 | -56.7 |
| B_001 | Lugana Superior | -37.0 | -28.1 | -19.8 | -47.9 | -38.7 | -30.3 | -47.9 | -38.6 | -29.8 | -70.0 | -57.7 | -46.2 |
| B_006 | San Diego | -19.1 | -14.8 | -10.8 | -24.6 | -20.1 | -16.1 | -22.2 | -17.7 | -13.4 | -35.6 | -29.5 | -24.0 |
| B_009 | Columbia River | 120.4 | 160.6 | 202.9 | 273.7 | 321.5 | 371.1 | 330.0 | 371.6 | 416.7 | 569.3 | 628.3 | 686.2 |
| B_012 | Tubarao Lagoon | -15.6 | -10.4 | -5.7 | -16.3 | -10.9 | -5.8 | -18.9 | -13.3 | -8.0 | -31.9 | -24.3 | -16.7 |
| B_017 | Rio Deseado | -17.0 | -13.2 | -9.6 | -27.1 | -23.1 | -19.4 | -26.4 | -22.3 | -18.5 | -37.0 | -31.4 | -26.3 |
| B_019 | Rio Chone | -3.1 | -1.8 | -0.5 | -4.5 | -3.1 | -1.8 | -3.2 | -1.7 | -0.3 | -5.1 | -3.2 | -1.4 |
| B_067 | Kaipara | -158.0 | -126.1 | -97.3 | -194.6 | -162.0 | -132.2 | -199.1 | -165.9 | -134.3 | -277.2 | -233.3 | -192.8 |
| B_071 | Gambia | 45.2 | 57.8 | 70.1 | 82.0 | 94.6 | 107.6 | 85.8 | 98.0 | 110.5 | 98.0 | 112.5 | 127.3 |
| B_073 | Cacheu | -26.5 | -21.2 | -16.3 | -32.3 | -26.9 | -22.0 | -33.3 | -27.7 | -22.5 | -46.4 | -39.0 | -32.3 |
| B_080 | Freetown | -31.3 | -22.6 | -14.7 | -37.5 | -28.6 | -20.5 | -35.8 | -26.8 | -18.2 | -51.9 | -40.2 | -29.1 |
| B_089 | Muni | -386.6 | -310.3 | -240.8 | -475.0 | -397.5 | -326.3 | -486.5 | -406.8 | -331.3 | -677.7 | -572.5 | -475.6 |
| B_090 | Gabon | -315.2 | -253.1 | -196.6 | -387.0 | -323.9 | -266.1 | -397.0 | -332.2 | -270.9 | -552.8 | -467.4 | -388.5 |
| B_091 | Zaire | -144.4 | -114.2 | -86.5 | -178.5 | -147.4 | -119.2 | -182.3 | -150.6 | -121.1 | -254.3 | -212.3 | -173.9 |
| B_093 | Inhambane | -22.6 | -18.1 | -14.0 | -27.4 | -22.8 | -18.6 | -28.6 | -23.9 | -19.5 | -39.7 | -33.5 | -27.8 |
| B_095 | Beira | -44.1 | -33.2 | -23.4 | -50.1 | -39.0 | -28.9 | -50.1 | -38.9 | -28.4 | -76.0 | -61.3 | -47.4 |
| B_104 | Miani Hor | -144.6 | -115.7 | -89.4 | -172.1 | -142.8 | -115.8 | -170.5 | -140.5 | -112.3 | -229.2 | -189.6 | -152.7 |
| B_111 | Hue | -36.2 | -28.5 | -21.3 | -45.1 | -37.1 | -29.8 | -45.9 | -37.7 | -30.0 | -64.4 | -53.6 | -43.6 |
| B_136 | Setubal | -50.8 | -40.7 | -31.6 | -62.3 | -52.1 | -42.6 | -64.6 | -54.0 | -43.9 | -90.5 | -76.7 | -63.7 |
| B_137 | Lisboa | -106.0 | -84.8 | -65.4 | -129.9 | -108.2 | -88.3 | -135.1 | -112.5 | -91.2 | -190.5 | -161.4 | -134.3 |
| B_139 | Gironde | -180.3 | -132.9 | -89.5 | -222.8 | -174.0 | -129.4 | -214.4 | -165.6 | -119.6 | -307.4 | -243.0 | -182.6 |
| B_140 | Loire | -38.4 | -22.9 | -8.3 | -38.7 | -23.1 | -8.8 | -25.1 | -9.7 | 5.1 | -64.0 | -44.5 | -25.8 |
| B_301 | Wilson | -10.0 | -8.0 | -6.1 | -12.6 | -10.2 | -8.4 | -12.4 | -10.4 | -8.4 | -17.2 | -14.6 | -12.1 |
| B_302 | St Paul | 9.1 | 11.1 | 13.2 | 9.6 | 11.7 | 13.8 | 12.8 | 15.0 | 17.2 | 18.0 | 20.2 | 22.7 |
| B_303 | Tweed River | -4.0 | -3.1 | -2.2 | -5.1 | -4.1 | -3.2 | -5.2 | -4.2 | -3.3 | -7.5 | -6.3 | -5.1 |
| B_304 | Richmond River | -5.4 | -3.8 | -2.3 | -7.2 | -5.5 | -4.0 | -7.3 | -5.6 | -3.9 | -11.3 | -9.1 | -7.0 |
| B_305 | Hastings River | -4.8 | -3.5 | -2.3 | -5.2 | -3.9 | -2.8 | -5.3 | -4.0 | -2.8 | -7.7 | -6.0 | -4.4 |
| B_306 | Shoalhaven River | -8.0 | -6.6 | -5.3 | -9.5 | -8.0 | -6.7 | -7.3 | -5.8 | -4.4 | -11.3 | -9.4 | -7.6 |
| B_307 | Macleay River | -2.9 | -1.4 | 0.1 | -1.9 | -0.4 | 1.0 | -1.8 | -0.3 | 1.2 | -3.3 | -1.3 | 0.6 |
| B_308 | Nambucca River | -2.1 | -1.6 | -1.1 | -2.4 | -1.9 | -1.4 | -2.5 | -1.9 | -1.4 | -3.5 | -2.8 | -2.1 |
| B_309 | Bellinger River | -1.2 | -0.8 | -0.4 | -0.9 | -0.5 | -0.1 | -1.1 | -0.7 | -0.3 | -1.3 | -0.8 | -0.4 |
| B_310 | Bega River | -0.8 | -0.7 | -0.5 | -1.1 | -0.9 | -0.7 | -0.8 | -0.6 | -0.4 | -1.3 | -1.0 | -0.8 |
| B_311 | Mawddach | -0.7 | -0.5 | -0.4 | -0.8 | -0.7 | -0.5 | -0.8 | -0.7 | -0.5 | -1.1 | -0.9 | -0.8 |
| B_312 | Exe | -3.8 | -3.1 | -2.4 | -4.6 | -3.8 | -3.1 | -4.8 | -3.9 | -3.2 | -6.6 | -5.6 | -4.6 |
| B_313 | Conwy | -1.1 | -0.8 | -0.6 | -1.2 | -1.0 | -0.8 | -1.3 | -1.0 | -0.8 | -1.8 | -1.5 | -1.2 |
| B_314 | Teign | -0.8 | -0.6 | -0.5 | -0.9 | -0.7 | -0.6 | -0.9 | -0.8 | -0.6 | -1.3 | -1.1 | -0.9 |
| B_315 | Thuan An | -18.5 | -14.0 | -9.9 | -23.5 | -18.9 | -14.7 | -23.5 | -18.8 | -14.3 | -33.4 | -27.1 | -21.4 |
| B_316 | Alsea | -1.5 | -1.1 | -0.7 | -1.7 | -1.3 | -0.9 | -1.6 | -1.1 | -0.7 | -2.1 | -1.5 | -1.0 |
| B_317 | Dyfi | -3.2 | -2.4 | -1.8 | -4.2 | -3.4 | -2.7 | -4.2 | -3.4 | -2.8 | -5.6 | -4.6 | -3.6 |
| B_318 | Kalutara | 2.1 | 2.7 | 3.3 | 3.9 | 4.6 | 5.4 | 4.3 | 5.1 | 5.8 | 5.5 | 6.3 | 7.2 |
| B_319 | Swan | -59.4 | -49.9 | -28.3 | -57.8 | -48.5 | -25.1 | -61.7 | -49.0 | -24.1 | -70.2 | -56.9 | -22.9 |

**Supplementary Table (S4)**

Table S4: The projected 10^th^, 50^th^, and 90^th^ percentiles of the time-averaged (2056-2065) change in total sediment volume exchange ($\boldsymbol{\Delta V}_{\boldsymbol{T}}$) at the 41 catchment-estuary-coastal systems under the 4 IPCC RCPs. Note: The time-averaged values of change in total sediment volume exchange were computed by averaging the projected 10^th^, 50^th^, and 90^th^ percentile values of $\boldsymbol{\Delta V}_{\boldsymbol{T}}$ over 2056-2065 period; negative signs denote sediment imported into the estuary

| Label | System Name | Time-averaged (2056-2065) values of the change in total sediment volume exchange ($\Delta V_{T}$) ($\times{10}^{6} m^{3}$) | | | | | | | | | | | |
| --- | --- | --- | --- | --- | --- | --- | --- | --- | --- | --- | --- | --- | --- |
|  |  | RCP 2.6 | | | RCP 4.5 | | | RCP 6.0 | | | RCP 8.5 | | |
|  |  | 10^th^ | 50^th^ | 90^th^ | 10^th^ | 50^th^ | 90^th^ | 10^th^ | 50^th^ | 90^th^ | 10^th^ | 50^th^ | 90^th^ |
| B_000 | Mar Muerto | -24.2 | -19.8 | -15.5 | -28.4 | -24.0 | -19.7 | -28.7 | -24.0 | -19.8 | -37.2 | -31.9 | -26.7 |
| B_001 | Lugana Superior | -20.0 | -16.1 | -12.3 | -24.1 | -20.1 | -16.4 | -24.1 | -19.9 | -16.1 | -32.0 | -27.2 | -22.6 |
| B_006 | San Diego | -10.1 | -8.1 | -6.3 | -12.1 | -10.2 | -8.3 | -11.2 | -9.2 | -7.4 | -16.0 | -13.7 | -11.4 |
| B_009 | Columbia River | 34.0 | 59.5 | 86.6 | 74.6 | 103.7 | 132.8 | 71.2 | 93.1 | 115.8 | 153.2 | 184.1 | 214.6 |
| B_012 | Tubarao Lagoon | -10.4 | -7.9 | -5.3 | -9.0 | -6.5 | -3.9 | -10.9 | -8.1 | -4.8 | -16.4 | -13.3 | -9.8 |
| B_017 | Rio Deseado | -10.1 | -8.4 | -6.7 | -13.4 | -11.6 | -9.9 | -12.6 | -10.9 | -9.1 | -15.5 | -13.2 | -11.1 |
| B_019 | Rio Chone | -2.2 | -1.6 | -1.0 | -2.6 | -2.0 | -1.4 | -2.2 | -1.5 | -0.9 | -3.0 | -2.2 | -1.5 |
| B_067 | Kaipara | -82.4 | -68.6 | -55.1 | -94.7 | -80.6 | -67.3 | -96.0 | -81.4 | -67.9 | -120.7 | -103.8 | -87.3 |
| B_071 | Gambia | 17.2 | 23.5 | 29.5 | 30.4 | 36.4 | 43.0 | 31.2 | 37.1 | 42.6 | 12.6 | 18.8 | 24.7 |
| B_073 | Cacheu | -13.8 | -11.5 | -9.2 | -15.6 | -13.3 | -11.0 | -16.0 | -13.6 | -11.3 | -20.3 | -17.5 | -14.7 |
| B_080 | Freetown | -18.0 | -14.2 | -10.5 | -20.2 | -16.3 | -12.6 | -20.3 | -16.3 | -12.5 | -26.7 | -22.3 | -17.8 |
| B_089 | Muni | -200.8 | -167.6 | -135.2 | -229.5 | -195.8 | -163.8 | -233.8 | -198.7 | -166.2 | -293.6 | -253.2 | -214.0 |
| B_090 | Gabon | -163.5 | -136.6 | -110.2 | -186.7 | -159.4 | -133.3 | -190.7 | -162.1 | -135.7 | -239.4 | -206.5 | -174.6 |
| B_091 | Zaire | -76.9 | -63.6 | -50.7 | -87.4 | -73.9 | -61.2 | -88.6 | -74.8 | -62.0 | -111.1 | -95.2 | -79.4 |
| B_093 | Inhambane | -11.7 | -9.8 | -7.8 | -13.2 | -11.2 | -9.3 | -13.8 | -11.7 | -9.8 | -17.2 | -14.8 | -12.5 |
| B_095 | Beira | -25.2 | -20.5 | -15.8 | -25.0 | -20.2 | -15.7 | -26.7 | -21.6 | -17.0 | -34.2 | -28.4 | -22.9 |
| B_104 | Miani Hor | -76.6 | -64.0 | -51.8 | -85.7 | -72.9 | -60.7 | -85.6 | -72.4 | -60.3 | -101.1 | -85.9 | -71.2 |
| B_111 | Hue | -19.1 | -15.7 | -12.3 | -22.2 | -18.7 | -15.4 | -22.6 | -19.0 | -15.6 | -28.2 | -24.1 | -20.0 |
| B_136 | Setubal | -26.5 | -22.1 | -17.9 | -30.1 | -25.6 | -21.4 | -31.0 | -26.3 | -21.9 | -38.7 | -33.4 | -28.2 |
| B_137 | Lisboa | -55.8 | -46.5 | -37.4 | -62.9 | -53.5 | -44.5 | -64.6 | -54.8 | -45.6 | -80.9 | -69.7 | -58.6 |
| B_139 | Gironde | -100.1 | -79.6 | -58.9 | -118.9 | -97.6 | -77.4 | -120.3 | -98.2 | -78.3 | -145.9 | -120.7 | -96.4 |
| B_140 | Loire | -25.4 | -18.2 | -11.1 | -26.0 | -19.0 | -12.4 | -21.7 | -15.0 | -8.5 | -33.4 | -25.6 | -17.8 |
| B_301 | Wilson | -5.1 | -4.2 | -3.4 | -6.1 | -5.0 | -4.2 | -5.7 | -4.9 | -4.1 | -7.5 | -6.5 | -5.5 |
| B_302 | St Paul | 3.2 | 4.1 | 5.0 | 2.9 | 3.8 | 4.8 | 3.4 | 4.3 | 5.2 | 4.8 | 5.7 | 6.8 |
| B_303 | Tweed River | -2.1 | -1.7 | -1.3 | -2.5 | -2.0 | -1.7 | -2.5 | -2.1 | -1.7 | -3.4 | -2.9 | -2.4 |
| B_304 | Richmond River | -3.0 | -2.3 | -1.5 | -3.5 | -2.8 | -2.1 | -3.6 | -2.8 | -2.0 | -5.4 | -4.5 | -3.6 |
| B_305 | Hastings River | -2.5 | -1.9 | -1.4 | -2.6 | -2.0 | -1.5 | -2.7 | -2.1 | -1.5 | -3.9 | -3.2 | -2.5 |
| B_306 | Shoalhaven River | -3.7 | -3.1 | -2.5 | -5.0 | -4.4 | -3.7 | -2.7 | -2.1 | -1.4 | -4.0 | -3.2 | -2.4 |
| B_307 | Macleay River | -1.5 | -0.8 | -0.1 | -1.2 | -0.5 | 0.2 | -1.2 | -0.4 | 0.3 | -2.9 | -2.0 | -1.1 |
| B_308 | Nambucca River | -1.1 | -0.9 | -0.6 | -1.2 | -1.0 | -0.7 | -1.2 | -1.0 | -0.7 | -1.7 | -1.4 | -1.1 |
| B_309 | Bellinger River | -0.6 | -0.4 | -0.3 | -0.5 | -0.3 | -0.1 | -0.6 | -0.4 | -0.3 | -0.8 | -0.6 | -0.4 |
| B_310 | Bega River | -0.4 | -0.3 | -0.3 | -0.4 | -0.3 | -0.2 | -0.3 | -0.2 | -0.1 | -0.6 | -0.5 | -0.4 |
| B_311 | Mawddach | -0.4 | -0.3 | -0.2 | -0.4 | -0.3 | -0.3 | -0.4 | -0.3 | -0.3 | -0.5 | -0.4 | -0.4 |
| B_312 | Exe | -2.0 | -1.7 | -1.4 | -2.3 | -1.9 | -1.6 | -2.3 | -2.0 | -1.6 | -2.9 | -2.5 | -2.1 |
| B_313 | Conwy | -0.6 | -0.5 | -0.4 | -0.6 | -0.5 | -0.4 | -0.6 | -0.5 | -0.4 | -0.8 | -0.7 | -0.6 |
| B_314 | Teign | -0.4 | -0.3 | -0.3 | -0.5 | -0.4 | -0.3 | -0.5 | -0.4 | -0.3 | -0.6 | -0.5 | -0.4 |
| B_315 | Thuan An | -9.9 | -7.9 | -6.0 | -11.8 | -9.8 | -7.9 | -11.9 | -9.8 | -7.9 | -14.8 | -12.4 | -10.0 |
| B_316 | Alsea | -0.9 | -0.7 | -0.5 | -1.0 | -0.8 | -0.6 | -0.9 | -0.7 | -0.5 | -1.0 | -0.8 | -0.6 |
| B_317 | Dyfi | -1.6 | -1.3 | -1.0 | -2.0 | -1.7 | -1.4 | -2.0 | -1.7 | -1.4 | -2.4 | -2.0 | -1.6 |
| B_318 | Kalutara | 0.4 | 0.8 | 1.2 | 1.1 | 1.5 | 1.9 | 1.2 | 1.6 | 2.0 | 1.5 | 1.9 | 2.5 |
| B_319 | Swan | -48.9 | -41.1 | -19.5 | -43.5 | -36.6 | -18.0 | -47.0 | -38.9 | -7.6 | -65.6 | -49.7 | -23.0 |

**Supplementary Table (S5)**

Table S5: The projected 10^th^, 50^th^, and 90^th^ percentiles of shoreline position change (relative to the present-day) along the inlet-affected coastlines adjacent to the 41 catchment-estuary-coastal systems by 2091-2100. Note: The 10^th^, 50^th^, and 90^th^ percentiles of shoreline position change correspond to the same percentiles of change in total sediment volume exchange ($\boldsymbol{\Delta V}_{\boldsymbol{T}}$) over 2091-2100 (Table S3). The sea-level rise driven shoreline retreat due to the Bruun effect is calculated for the change in global mean sea level by 2091-2100 (relative to present-day)

| Label | System Name | Projected change in shoreline position by 2091-2100, relative to present-day (m) | | | | | | | | | | | |
| --- | --- | --- | --- | --- | --- | --- | --- | --- | --- | --- | --- | --- | --- |
|  |  | RCP 2.6 | | | RCP 4.5 | | | RCP 6.0 | | | RCP 8.5 | | |
|  |  | 10^th^ | 50^th^ | 90^th^ | 10^th^ | 50^th^ | 90^th^ | 10^th^ | 50^th^ | 90^th^ | 10^th^ | 50^th^ | 90^th^ |
| B_000 | Mar Muerto | -129 | -108 | -90 | -164 | -143 | -124 | -167 | -145 | -126 | -238 | -210 | -184 |
| B_001 | Lugana Superior | -126 | -120 | -120 | -165 | -161 | -159 | -167 | -163 | -161 | -243 | -236 | -233 |
| B_006 | San Diego | -104 | -92 | -84 | -135 | -124 | -115 | -126 | -115 | -106 | -197 | -181 | -168 |
| B_009 | Columbia River | 81 | 111 | 140 | 221 | 257 | 295 | 276 | 306 | 337 | 491 | 535 | 577 |
| B_012 | Tubarao Lagoon | -148 | -116 | -89 | -163 | -130 | -99 | -184 | -150 | -118 | -301 | -254 | -207 |
| B_017 | Rio Deseado | -77 | -79 | -84 | -113 | -116 | -120 | -113 | -116 | -121 | -160 | -163 | -168 |
| B_019 | Rio Chone | -59 | -56 | -57 | -81 | -79 | -78 | -72 | -69 | -69 | -108 | -104 | -103 |
| B_067 | Kaipara | -183 | -159 | -140 | -228 | -205 | -184 | -234 | -210 | -188 | -327 | -295 | -267 |
| B_071 | Gambia | 21 | 28 | 27 | 85 | 88 | 92 | 91 | 93 | 93 | 63 | 64 | 61 |
| B_073 | Cacheu | -153 | -168 | -192 | -199 | -218 | -237 | -205 | -223 | -246 | -293 | -315 | -342 |
| B_080 | Freetown | -202 | -199 | -207 | -256 | -257 | -260 | -254 | -254 | -260 | -368 | -366 | -371 |
| B_089 | Muni | -387 | -341 | -306 | -483 | -439 | -399 | -495 | -449 | -409 | -695 | -632 | -579 |
| B_090 | Gabon | -311 | -273 | -243 | -388 | -351 | -317 | -398 | -359 | -325 | -558 | -506 | -461 |
| B_091 | Zaire | -231 | -202 | -178 | -290 | -261 | -235 | -297 | -267 | -241 | -417 | -376 | -341 |
| B_093 | Inhambane | -83 | -74 | -67 | -103 | -94 | -86 | -107 | -98 | -90 | -150 | -137 | -126 |
| B_095 | Beira | -293 | -272 | -262 | -354 | -335 | -320 | -358 | -338 | -325 | -533 | -505 | -485 |
| B_104 | Miani Hor | -273 | -234 | -202 | -331 | -292 | -257 | -329 | -289 | -253 | -447 | -393 | -346 |
| B_111 | Hue | -188 | -195 | -211 | -244 | -254 | -266 | -250 | -259 | -274 | -356 | -366 | -383 |
| B_136 | Setubal | -156 | -130 | -108 | -193 | -167 | -143 | -200 | -173 | -147 | -280 | -245 | -213 |
| B_137 | Lisboa | -215 | -178 | -146 | -265 | -228 | -194 | -275 | -236 | -200 | -389 | -338 | -292 |
| B_139 | Gironde | -305 | -260 | -224 | -385 | -339 | -300 | -376 | -331 | -292 | -540 | -479 | -426 |
| B_140 | Loire | -193 | -159 | -133 | -216 | -185 | -157 | -174 | -142 | -115 | -343 | -303 | -268 |
| B_301 | Wilson | -45 | -40 | -37 | -57 | -52 | -49 | -57 | -53 | -49 | -80 | -74 | -70 |
| B_302 | St Paul | 6 | 6 | 3 | -4 | -6 | -7 | 10 | 9 | 6 | 12 | 8 | 4 |
| B_303 | Tweed River | -110 | -109 | -113 | -144 | -144 | -146 | -147 | -147 | -150 | -213 | -212 | -215 |
| B_304 | Richmond River | -44 | -40 | -38 | -59 | -55 | -52 | -60 | -56 | -53 | -90 | -85 | -81 |
| B_305 | Hastings River | -52 | -50 | -50 | -63 | -61 | -60 | -64 | -62 | -62 | -93 | -90 | -89 |
| B_306 | Shoalhaven River | -86 | -82 | -80 | -106 | -102 | -99 | -90 | -86 | -84 | -136 | -131 | -127 |
| B_307 | Macleay River | -40 | -35 | -31 | -40 | -35 | -31 | -40 | -35 | -31 | -62 | -56 | -51 |
| B_308 | Nambucca River | -35 | -36 | -39 | -43 | -46 | -48 | -45 | -47 | -50 | -64 | -67 | -70 |
| B_309 | Bellinger River | -26 | -29 | -34 | -31 | -35 | -39 | -33 | -37 | -42 | -47 | -51 | -57 |
| B_310 | Bega River | -23 | -24 | -26 | -31 | -32 | -33 | -27 | -28 | -29 | -41 | -42 | -44 |
| B_311 | Mawddach | -71 | -87 | -108 | -94 | -113 | -132 | -97 | -116 | -137 | -140 | -163 | -189 |
| B_312 | Exe | -102 | -97 | -95 | -128 | -124 | -120 | -132 | -127 | -124 | -185 | -178 | -174 |
| B_313 | Conwy | -51 | -56 | -64 | -66 | -72 | -78 | -68 | -73 | -81 | -97 | -104 | -113 |
| B_314 | Teign | -42 | -46 | -53 | -54 | -60 | -65 | -55 | -61 | -68 | -79 | -86 | -94 |
| B_315 | Thuan An | -157 | -153 | -154 | -205 | -202 | -201 | -208 | -204 | -204 | -297 | -290 | -289 |
| B_316 | Alsea | -21 | -24 | -28 | -27 | -31 | -35 | -27 | -31 | -35 | -39 | -43 | -49 |
| B_317 | Dyfi | -54 | -56 | -60 | -72 | -74 | -77 | -74 | -76 | -80 | -103 | -105 | -108 |
| B_318 | Kalutara | -29 | -37 | -47 | -34 | -43 | -51 | -33 | -42 | -52 | -52 | -63 | -75 |
| B_319 | Swan | -182 | -180 | -161 | -201 | -203 | -177 | -211 | -206 | -180 | -268 | -267 | -227 |

**Supplementary Table (S6)**

Table S6: The projected 10^th^, 50^th^, and 90^th^ percentiles of shoreline position change (relative to the present-day) along the inlet-affected coastlines adjacent to the 41 catchment-estuary-coastal systems by 2056-2065. Note: The 10^th^, 50^th^, and 90^th^ percentiles of shoreline position change correspond to the same percentiles of change in total sediment volume exchange ($\boldsymbol{\Delta V}_{\boldsymbol{T}}$) over 2056-2065 (Table S4). The sea-level rise driven shoreline retreat due to the Bruun effect is calculated for the change in global mean sea level by 2056-2065 (relative to present-day)

| Label | System Name | Projected change in shoreline position by 2056-2065, relative to present-day (m) | | | | | | | | | | | |
| --- | --- | --- | --- | --- | --- | --- | --- | --- | --- | --- | --- | --- | --- |
|  |  | RCP 2.6 | | | RCP 4.5 | | | RCP 6.0 | | | RCP 8.5 | | |
|  |  | 10^th^ | 50^th^ | 90^th^ | 10^th^ | 50^th^ | 90^th^ | 10^th^ | 50^th^ | 90^th^ | 10^th^ | 50^th^ | 90^th^ |
| B_000 | Mar Muerto | -69 | -60 | -51 | -81 | -72 | -64 | -82 | -73 | -64 | -106 | -96 | -85 |
| B_001 | Lugana Superior | -70 | -67 | -67 | -82 | -81 | -81 | -84 | -83 | -80 | -110 | -109 | -107 |
| B_006 | San Diego | -56 | -51 | -47 | -66 | -62 | -58 | -64 | -59 | -54 | -88 | -83 | -78 |
| B_009 | Columbia River | 11 | 33 | 54 | 49 | 72 | 95 | 44 | 60 | 78 | 119 | 142 | 167 |
| B_012 | Tubarao Lagoon | -96 | -80 | -64 | -88 | -72 | -56 | -103 | -86 | -64 | -150 | -132 | -110 |
| B_017 | Rio Deseado | -45 | -46 | -48 | -55 | -57 | -59 | -55 | -57 | -58 | -69 | -71 | -73 |
| B_019 | Rio Chone | -37 | -35 | -36 | -43 | -43 | -43 | -41 | -40 | -39 | -54 | -54 | -52 |
| B_067 | Kaipara | -97 | -87 | -78 | -111 | -102 | -93 | -113 | -103 | -93 | -143 | -132 | -120 |
| B_071 | Gambia | -11 | -6 | -5 | 16 | 17 | 19 | 15 | 15 | 18 | -53 | -56 | -55 |
| B_073 | Cacheu | -85 | -92 | -102 | -97 | -107 | -117 | -101 | -111 | -118 | -129 | -142 | -151 |
| B_080 | Freetown | -116 | -115 | -117 | -131 | -133 | -136 | -135 | -137 | -135 | -175 | -179 | -179 |
| B_089 | Muni | -205 | -185 | -168 | -234 | -216 | -199 | -240 | -221 | -201 | -302 | -281 | -258 |
| B_090 | Gabon | -164 | -148 | -133 | -187 | -172 | -158 | -193 | -176 | -160 | -243 | -225 | -206 |
| B_091 | Zaire | -125 | -112 | -101 | -142 | -130 | -119 | -145 | -133 | -120 | -183 | -169 | -154 |
| B_093 | Inhambane | -44 | -40 | -36 | -50 | -46 | -43 | -52 | -48 | -44 | -65 | -61 | -56 |
| B_095 | Beira | -168 | -158 | -153 | -175 | -168 | -163 | -186 | -179 | -169 | -238 | -231 | -222 |
| B_104 | Miani Hor | -146 | -129 | -114 | -164 | -148 | -132 | -165 | -148 | -132 | -197 | -178 | -159 |
| B_111 | Hue | -104 | -107 | -113 | -119 | -125 | -132 | -124 | -130 | -133 | -157 | -165 | -170 |
| B_136 | Setubal | -82 | -71 | -60 | -93 | -82 | -72 | -96 | -84 | -73 | -120 | -107 | -94 |
| B_137 | Lisboa | -114 | -97 | -82 | -128 | -112 | -97 | -132 | -115 | -99 | -166 | -147 | -128 |
| B_139 | Gironde | -171 | -151 | -133 | -201 | -183 | -166 | -205 | -186 | -167 | -252 | -231 | -208 |
| B_140 | Loire | -122 | -106 | -92 | -129 | -116 | -104 | -117 | -105 | -91 | -169 | -155 | -139 |
| B_301 | Wilson | -23 | -22 | -20 | -28 | -26 | -24 | -27 | -26 | -24 | -35 | -33 | -31 |
| B_302 | St Paul | -7 | -7 | -8 | -11 | -12 | -14 | -10 | -12 | -12 | -11 | -13 | -14 |
| B_303 | Tweed River | -61 | -60 | -62 | -70 | -71 | -72 | -72 | -73 | -72 | -95 | -97 | -97 |
| B_304 | Richmond River | -25 | -23 | -22 | -29 | -28 | -26 | -29 | -28 | -26 | -42 | -40 | -38 |
| B_305 | Hastings River | -28 | -27 | -27 | -31 | -31 | -30 | -32 | -32 | -31 | -44 | -44 | -43 |
| B_306 | Shoalhaven River | -43 | -41 | -40 | -55 | -53 | -52 | -38 | -36 | -34 | -52 | -51 | -49 |
| B_307 | Macleay River | -22 | -19 | -17 | -21 | -19 | -17 | -22 | -20 | -17 | -38 | -35 | -32 |
| B_308 | Nambucca River | -19 | -20 | -21 | -21 | -23 | -24 | -22 | -23 | -24 | -29 | -31 | -32 |
| B_309 | Bellinger River | -15 | -16 | -18 | -15 | -17 | -19 | -17 | -19 | -20 | -22 | -25 | -27 |
| B_310 | Bega River | -13 | -13 | -13 | -13 | -14 | -14 | -12 | -13 | -13 | -19 | -20 | -20 |
| B_311 | Mawddach | -41 | -48 | -57 | -46 | -55 | -65 | -48 | -58 | -65 | -62 | -74 | -83 |
| B_312 | Exe | -56 | -53 | -52 | -62 | -61 | -60 | -65 | -63 | -61 | -82 | -81 | -78 |
| B_313 | Conwy | -29 | -31 | -34 | -32 | -36 | -39 | -34 | -37 | -39 | -43 | -47 | -50 |
| B_314 | Teign | -24 | -26 | -29 | -26 | -29 | -32 | -28 | -31 | -33 | -35 | -39 | -42 |
| B_315 | Thuan An | -87 | -85 | -85 | -102 | -101 | -102 | -105 | -104 | -102 | -131 | -132 | -130 |
| B_316 | Alsea | -12 | -13 | -15 | -14 | -15 | -17 | -14 | -16 | -17 | -17 | -20 | -22 |
| B_317 | Dyfi | -30 | -30 | -32 | -35 | -37 | -38 | -36 | -38 | -38 | -44 | -47 | -48 |
| B_318 | Kalutara | -21 | -23 | -27 | -20 | -24 | -28 | -21 | -25 | -28 | -27 | -33 | -36 |
| B_319 | Swan | -133 | -126 | -93 | -128 | -124 | -98 | -137 | -131 | -77 | -186 | -168 | -125 |

**Supplementary Table (S7)**

Table S7: Comparison of projected shoreline position change by 2100 (relative to present-day) with the results of Vousdoukas et al. (2020)^13^’s global assessment of shoreline retreat/progradation along sandy coasts. Positive and negative values indicate shoreline progradation and retreat, respectively. The 50^th^ percentile values of the projected shoreline position change by Vousdoukas et al. (2020)^13^ in the vicinities of the 41 catchment-estuary-coastal systems considered here are used in this comparison. Projection variability of G-SMIC application indicates the difference between 90^th^ and 10^th^ percentile values of the projected shoreline position change

| Label | System Name | Region/ Country | Projected median shoreline position change (m) by 2100 relative to present-day | | | | | |
| --- | --- | --- | --- | --- | --- | --- | --- | --- |
|  |  |  | G-SMIC application | | | | Vousdoukas et al. (2020)^13^ | |
|  |  |  | RCP 4.5 | | RCP 8.5 | | RCP 4.5 | RCP 8.5 |
|  |  |  | 50^th^ percentile | Projection variability | 50^th^ percentile | Projection variability |  |  |
| B_000 | Mar Muerto | Central America | -143 | 40 | -210 | 55 | -100 | -150 |
| B_001 | Lugana |  | -161 | 7 | -236 | 10 | -100 | -150 |
| B_006 | San Diego | West USA | -124 | 21 | -181 | 29 | -50 | -100 |
| B_009 | Columbia River | Northwest USA | 257 | 73 | 535 | 85 | -50 | -50 |
| B_012 | Tubarao | East-mid South America | -130 | 64 | -254 | 94 | -100 | -150 |
| B_017 | Rio Deseado | East-low. South America | -116 | 7 | -163 | 8 | -100 | -150 |
| B_019 | Rio Chone | Northwest South America | -79 | 3 | -104 | 4 | -100 | -100 |
| B_067 | Kaipara | New Zealand | -205 | 44 | -295 | 60 | -200 | -200 |
| B_071 | Gambia | Western Africa | 88 | 7 | 64 | 2 | -200 | -200 |
| B_073 | Cacheu |  | -218 | 38 | -315 | 49 | -200 | -200 |
| B_080 | Freetown |  | -257 | 3 | -366 | 3 | -200 | -200 |
| B_089 | Muni |  | -439 | 84 | -632 | 116 | -200 | -200 |
| B_090 | Gabon |  | -351 | 71 | -506 | 97 | -200 | -200 |
| B_091 | Zaire |  | -261 | 55 | -376 | 76 | -200 | -200 |
| B_093 | Inhambane | Eastern Africa | -94 | 17 | -137 | 24 | -150 | -150 |
| B_095 | Beira |  | -335 | 34 | -505 | 48 | -150 | -150 |
| B_104 | Miani Hor | Central Asia | -292 | 74 | -393 | 101 | -150 | -200 |
| B_111 | Hue | Mid-Vietnam | -254 | 22 | -366 | 27 | -200 | -200 |
| B_136 | Setubal | Southwest of Portugal | -167 | 50 | -245 | 68 | -100 | -150 |
| B_137 | Lisboa |  | -228 | 71 | -338 | 97 | -100 | -150 |
| B_139 | Gironde | Northwest of France | -339 | 85 | -479 | 114 | -200 | -200 |
| B_140 | Loire |  | -185 | 60 | -303 | 75 | -200 | -200 |
| B_301 | Wilson | Western Australia | -52 | 8 | -74 | 10 | -50 | -50 |
| B_302 | St Paul | West Africa | -6 | 3 | 8 | 8 | -50 | -50 |
| B_303 | Tweed River | NSW (Australia) | -144 | 2 | -212 | 1 | -50 | -100 |
| B_304 | Richmond River |  | -55 | 6 | -85 | 9 | -50 | -100 |
| B_305 | Hastings River |  | -61 | 2 | -90 | 4 | -50 | -100 |
| B_306 | Shoalhaven River |  | -102 | 7 | -131 | 9 | -50 | -100 |
| B_307 | Macleay River |  | -35 | 9 | -56 | 12 | -50 | -100 |
| B_308 | Nambucca |  | -46 | 5 | -67 | 6 | -50 | -100 |
| B_309 | Bellinger River |  | -35 | 8 | -51 | 10 | -50 | -100 |
| B_310 | Bega River |  | -32 | 1 | -42 | 3 | -50 | -100 |
| B_311 | Mawddach | West and south of UK | -113 | 38 | -163 | 49 | -100 | -100 |
| B_312 | Exe |  | -124 | 8 | -178 | 11 | -100 | -100 |
| B_313 | Conwy |  | -72 | 12 | -104 | 16 | -100 | -100 |
| B_314 | Teign |  | -60 | 11 | -86 | 15 | -100 | -100 |
| B_315 | Thuan An | Mid-Vietnam | -202 | 4 | -290 | 7 | -200 | -200 |
| B_316 | Alsea | West of USA | -31 | 8 | -43 | 10 | -50 | -50 |
| B_317 | Dyfi | West of UK | -74 | 5 | -105 | 6 | -100 | -100 |
| B_318 | Kalutara | West of Sri Lanka | -43 | 17 | -63 | 23 | -100 | -150 |
| B_319 | Swan | Western Australia | -203 | 24 | -267 | 40 | -150 | -200 |

**Supplementary Table (S8)**

Table S8: Comparison of the projected median change in shoreline position along the inlet-interrupted coastlines adjacent to the 41 catchment-estuary-coastal systems by 2091-2100 (relative to present-day), obtained from G-SMIC application and the Bruun rule only. Positive and negative values indicate shoreline progradation and retreat, respectively. Note: The sea-level rise driven shoreline retreat due to the Bruun effect is calculated for the median change in global mean sea level by 2091-2100 (relative to present-day).

| Label | System Name | Projected median change in shoreline position by 2091-2100, relative to present-day (m) | | | | | | | |
| --- | --- | --- | --- | --- | --- | --- | --- | --- | --- |
|  |  | RCP 2.6 | | RCP 4.5 | | RCP 6.0 | | RCP 8.5 | |
|  |  | G-SMIC | Bruun rule only | G-SMIC | Bruun rule only | G-SMIC | Bruun rule only | G-SMIC | Bruun rule only |
| B_000 | Mar Muerto | -108 | -19 | -143 | -25 | -145 | -25 | -210 | -36 |
| B_001 | Lugana Superior | -120 | -62 | -161 | -81 | -163 | -83 | -236 | -116 |
| B_006 | San Diego | -92 | -31 | -124 | -40 | -115 | -41 | -181 | -58 |
| B_009 | Columbia River | 111 | -50 | 257 | -65 | 306 | -66 | 535 | -93 |
| B_012 | Tubarao Lagoon | -116 | -35 | -130 | -46 | -150 | -47 | -254 | -66 |
| B_017 | Rio Deseado | -79 | -50 | -116 | -65 | -116 | -66 | -163 | -93 |
| B_019 | Rio Chone | -56 | -41 | -79 | -54 | -69 | -55 | -104 | -78 |
| B_067 | Kaipara | -159 | -35 | -205 | -46 | -210 | -47 | -295 | -66 |
| B_071 | Gambia | 28 | -124 | 88 | -161 | 93 | -165 | 64 | -233 |
| B_073 | Cacheu | -168 | -124 | -218 | -161 | -223 | -165 | -315 | -233 |
| B_080 | Freetown | -199 | -124 | -257 | -161 | -254 | -165 | -366 | -233 |
| B_089 | Muni | -341 | -83 | -439 | -108 | -449 | -110 | -632 | -155 |
| B_090 | Gabon | -273 | -62 | -351 | -81 | -359 | -83 | -506 | -116 |
| B_091 | Zaire | -202 | -50 | -261 | -65 | -267 | -66 | -376 | -93 |
| B_093 | Inhambane | -74 | -19 | -94 | -25 | -98 | -25 | -137 | -36 |
| B_095 | Beira | -272 | -124 | -335 | -161 | -338 | -165 | -505 | -233 |
| B_104 | Miani Hor | -234 | -41 | -292 | -54 | -289 | -55 | -393 | -78 |
| B_111 | Hue | -195 | -124 | -254 | -161 | -259 | -165 | -366 | -233 |
| B_136 | Setubal | -130 | -14 | -167 | -18 | -173 | -18 | -245 | -26 |
| B_137 | Lisboa | -178 | -17 | -228 | -22 | -236 | -22 | -338 | -31 |
| B_139 | Gironde | -260 | -83 | -339 | -108 | -331 | -110 | -479 | -155 |
| B_140 | Loire | -159 | -83 | -185 | -108 | -142 | -110 | -303 | -155 |
| B_301 | Wilson | -40 | -12 | -52 | -16 | -53 | -17 | -74 | -23 |
| B_302 | St Paul | 6 | -50 | -6 | -65 | 9 | -66 | 8 | -93 |
| B_303 | Tweed River | -109 | -62 | -144 | -81 | -147 | -83 | -212 | -116 |
| B_304 | Richmond River | -40 | -21 | -55 | -27 | -56 | -28 | -85 | -39 |
| B_305 | Hastings River | -50 | -28 | -61 | -36 | -62 | -37 | -90 | -52 |
| B_306 | Shoalhaven River | -82 | -31 | -102 | -40 | -86 | -41 | -131 | -58 |
| B_307 | Macleay River | -35 | -25 | -35 | -32 | -35 | -33 | -56 | -47 |
| B_308 | Nambucca River | -36 | -25 | -46 | -32 | -47 | -33 | -67 | -47 |
| B_309 | Bellinger River | -29 | -25 | -35 | -32 | -37 | -33 | -51 | -47 |
| B_310 | Bega River | -24 | -15 | -32 | -19 | -28 | -19 | -42 | -27 |
| B_311 | Mawddach | -87 | -83 | -113 | -108 | -116 | -110 | -163 | -155 |
| B_312 | Exe | -97 | -41 | -124 | -54 | -127 | -55 | -178 | -78 |
| B_313 | Conwy | -56 | -41 | -72 | -54 | -73 | -55 | -104 | -78 |
| B_314 | Teign | -46 | -35 | -60 | -46 | -61 | -47 | -86 | -66 |
| B_315 | Thuan An | -153 | -83 | -202 | -108 | -204 | -110 | -290 | -155 |
| B_316 | Alsea | -24 | -21 | -31 | -27 | -31 | -28 | -43 | -39 |
| B_317 | Dyfi | -56 | -35 | -74 | -46 | -76 | -47 | -105 | -66 |
| B_318 | Kalutara | -37 | -50 | -43 | -65 | -42 | -66 | -63 | -93 |
| B_319 | Swan | -180 | -83 | -203 | -108 | -206 | -110 | -267 | -155 |

**References**

1. Hinkel, J. *et al.* A global analysis of erosion of sandy beaches and sea-level rise: An application of DIVA. *Glob. Planet. Change* **111**, 150–158 (2013).

2. Manning, A. J. *TR167 – Enhanced UK Estuaries database: explanatory notes and metadata. HR Wallingford Report DDY0427-RT002-R02-00*. (2012).

3. Office of Environment and Heritage-New South Wales. Estuaries of NSW. *Retrieved from https://www.environment.nsw.gov.au/topics/water/estuaries/estuaries-of-nsw* (2017).

4. Ranasinghe, R., Duong, T. M., Uhlenbrook, S., Roelvink, D. & Stive, M. Climate-change impact assessment for inlet-interrupted coastlines. *Nat. Clim. Chang.* **3**, 83–87 (2013).

5. Bamunawala, J. *et al.* Probabilistic Application of an Integrated Catchment-Estuary-Coastal System Model to Assess the Evolution of Inlet-Interrupted Coasts Over the 21st Century. *Front. Mar. Sci.* **7**, 1104 (2020).

6. Norwegian Water Resources and Energy Directorate & Liberian Hydrological Service: Ministry of Lands Mines and Energy. Liberian Hydrological Services. http://lhsliberia.com/ (2019).

7. Liberian Hydrological Service: Ministry of Lands Mines and Energy & Norwegian Water Resources and Energy Directorate. *Liberia River Basins: Drainage Divisions and river Basin Boundaries*. (2016).

8. Bamunawala, J. A holistic modelling approach to simulate catchment-estuary-coastal system behaviour at macro-time scales. (University of Twente, 2020). doi:10.3990/1.9789036549882.

9. Syvitski, J. P. M. M. & Milliman, J. D. Geology, geography, and humans battle for dominance over the delivery of fluvial sediment to the coastal ocean. *J. Geol.* **115**, 1–19 (2007).

10. Farr, T. G. *et al.* The Shuttle Radar Topography Mission. *Rev. Geophys.* **45**, RG2004 (2007).

11. Wildlife Conservation Society (WCS) and Center for International Earth Science Information Network (CIESIN) - Columbia University., Wildlife Conservation Society-WCS & Center for International Earth Science Information Network-CIESIN-Columbia University. *Last of the Wild Project, Version 2, 2005 (LWP-2): Global Human Footprint Dataset (Geographic)*. *Palisades, NY: NASA Socioeconomic Data and Applications Center (SEDAC)* (2005). doi:http://dx.doi.org/10.7927/H4M61H5F.

12. Athanasiou, P. *et al.* Global distribution of nearshore slopes with implications for coastal retreat. *Earth Syst. Sci. Data* **11**, 1515–1529 (2019).

13. Vousdoukas, M. I. *et al.* Sandy coastlines under threat of erosion. *Nat. Clim. Chang.* **10**, 260–263 (2020).

14. Luijendijk, A. *et al.* The State of the World’s Beaches. *Sci. Rep.* **8**, 6641 (2018).
